# Steady-State Metabolite Concentrations Reflect a Balance between Maximizing Enzyme Efficiency and Minimizing Total Metabolite Load

**DOI:** 10.1371/journal.pone.0075370

**Published:** 2013-09-26

**Authors:** Naama Tepper, Elad Noor, Daniel Amador-Noguez, Hulda S. Haraldsdóttir, Ron Milo, Josh Rabinowitz, Wolfram Liebermeister, Tomer Shlomi

**Affiliations:** 1 Department of Computer Science, Technion–IIT, Haifa, Israel; 2 Department of Plant Sciences, Weizmann Institute of Science, Rehovot, Israel; 3 Chemistry and Integrative Genomics, Princeton University, Princeton, New Jersey, United States of America; 4 Center for Systems Biology, University of Iceland, Reykjavik, Iceland; 5 Institut fürBiochemie, Charite -Universitätsmedizin Berlin, Berlin, Germany; Tata Institute of Fundamental Research, India

## Abstract

Steady-state metabolite concentrations in a microorganism typically span several orders of magnitude. The underlying principles governing these concentrations remain poorly understood. Here, we hypothesize that observed variation can be explained in terms of a compromise between factors that favor minimizing metabolite pool sizes (e.g. limited solvent capacity) and the need to effectively utilize existing enzymes. The latter requires adequate thermodynamic driving force in metabolic reactions so that forward flux substantially exceeds reverse flux. To test this hypothesis, we developed a method, metabolic tug-of-war (mTOW), which computes steady-state metabolite concentrations in microorganisms on a genome-scale. mTOW is shown to explain up to 55% of the observed variation in measured metabolite concentrations in *E. coli* and *C. acetobutylicum* across various growth media. Our approach, based strictly on first thermodynamic principles, is the first method that successfully predicts high-throughput metabolite concentration data in bacteria across conditions.

## Introduction

Cellular metabolism involves the joint activity of hundreds of enzyme-catalyzed biochemical reaction A system-wide understanding of cellular metabolism requires the quantification of both fluxes through these reactions and the concentrations of the corresponding metabolites. In recent years, mass spectrometry has become a popular tool for high-throughput measurements of metabolite concentrations. In combination with isotope tracing, mass spectrometry can also measure metabolic fluxes [1-3]. The application of this approach and others has revealed that both metabolite concentrations and fluxes span several orders of magnitude and significantly vary across microorganisms and growth conditions [4-6].

For steady-state systems, substantial insight into metabolic reaction rates can be achieved through constraints imposed by the law of mass balance under a steady-state assumption: for each internal metabolite, total influx must equal total efflux. Systems level application of this constraint, typically referred to as Flux Balance Analysis (FBA) [7], requires knowledge only of metabolic network stoichiometry, without requiring data on enzyme kinetic constants. Nevertheless, it has shown substantial predictive power for both fluxes and other phenotypes [2,8,9]. Accordingly, FBA has become a widely used tool in bioengineering [10-13].

In contrast to the success of FBA in predicting fluxes, there is no comparable tool for explaining and predicting metabolite concentrations in cell-wide setups. Explicitly modeling metabolite concentrations would require a systematic understanding of in vivo enzyme kinetics, which is currently lacking, despite ongoing progress on modeling metabolic systems using simplified rate equations and parameter estimation techniques [14-18].

An alternative approach involves extending FBA to genome-scale modeling of metabolite concentrations by accounting for thermodynamic considerations [19-24] (without modeling enzyme kinetic effects). These methods capitalized on the fact that the net flux direction depends on the thermodynamic driving force, −*ΔG*', which is calculated according to the equation:

$$\Delta G'=\Delta G{'}^{0}+RT\ln Q$$ (1)

Where *Q* is the ratio of the chemical activities of products and reactants within the compartment where the reaction is occurring, *R* is the gas constant, *T* is the temperature, and *ΔG*'^0^ is the standard reaction Gibbs energy (determined by the change in Gibbs energy of formation between products and substrates at standard concentrations). Throughout this work we assume the cytoplasm is a dilute aqueous solution (and therefore the metabolite chemical activities are equal to their absolute concentrations [25]). Extended FBA methods implementing thermodynamic constraints on reaction directionality were shown to improve flux predictions [19,20]. However, incorporating the second law of thermodynamics per-se into FBA (i.e. constraining *ΔG*'<0 for flux-carrying reactions) is insufficient for effectively predicting metabolite concentrations, as for most metabolites, a wide range of possible concentrations satisfies all network thermodynamic constraints [19].

Schuster and Heinrich [26] suggested the principle of minimization of intermediate concentrations to identify biologically plausible metabolite concentrations within the space of thermodynamically feasible solutions (see also 27). The maintenance of low metabolite concentrations is due to limitations on intracellular solvent capacity, osmotic pressure, and to facilitate rapid temporal responses to parameter changes [28]. It further serves to reduce cross-talk between metabolic pathways that may arise at high metabolite concentrations due to promiscuous activity of enzymes (i.e., enzymes acting non-specifically on substrates other than their natural ones) [29,30]. Indeed, recent studies have shown that the total intracellular levels of metabolites is limited to around 10 gr/L and 300 mM which amounts to 3% of the total cellular dry weight in *E. coli* [4,31,32] (see Supp. Material). Taking this optimality consideration into account, Schuster and Heinrich have analyzed the metabolite concentrations in key metabolic pathways in human erythrocytes, showing that their prediction corresponds qualitatively to experimental data [26].

**Figure 1:**
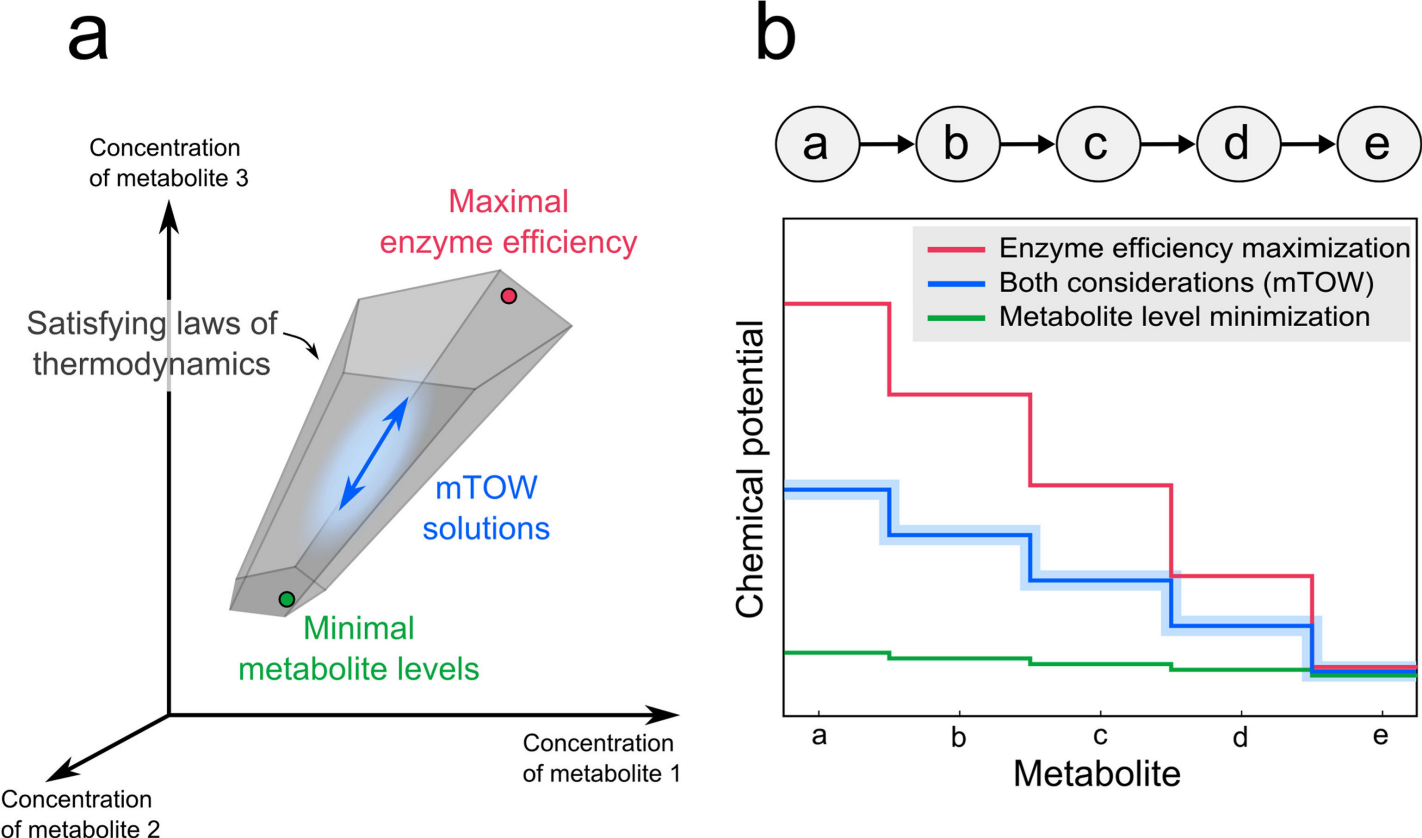
Steady-state metabolite concentrations reflect a balance between minimizing metabolite levels versus maximizing enzyme efficiency. (a) The second law of thermodynamics alone is insufficient to uniquely determine metabolite concentrations, resulting in a space of possible metabolite levels. Two factors “pull” metabolite levels within this space in different directions: (I) a limitation of the solvent capacity and osmotic pressure that tends to drive metabolite concentrations down; (II) the cost associated with the production of enzymes that drives up ratios of reactant-to-product concentrations, and thereby total metabolite concentrations. (b) A toy example of a linear pathway. The chemical potential of each metabolite in the pathway depends on its concentration and intrinsic energy of formation. A drop in metabolite chemical potential at every step is a thermodynamic requirement for the pathway to carry flux. Considering only the objective of maximizing enzyme efficiency (i.e., forward flux per enzyme), it is desirable to achieve a large drop in chemical potential at each step, but this requires unrealistically high total metabolite concentrations (red line). On the other hand, solutions that minimize total metabolite concentrations result in small drops in chemical potential (with many reactions close to equilibrium), leading to inefficient enzyme utilization (most capacity lost to backwards flux) (green line). This in turn requires unrealistically high enzyme levels to produce the required metabolic flux. mTOW balances both factors (blue line.).

Here, we suggest that steady-state metabolite concentrations reflect a compromise between limiting the total concentration of intermediate metabolites (“metabolite load”; as previously suggested by Schuster and Heinrich) and maintaining sufficient thermodynamic driving force such that enzymes are utilized efficiently, with most flux in the forward direction. By maintaining adequate forward driving force for all reactions, the enzyme concentrations required to support the necessary net metabolic flux is reduced. Thus, steady-state metabolite concentrations reflect a balance between minimizing metabolite levels and maximizing enzyme efficiency (Figure 1). Minimizing enzyme levels is important, due to space restrictions in the cell and the costs of protein production and maintenance [33].

We formalize the trade-off between metabolite and enzyme levels in a computational framework, which we term metabolic Tug-of-War (mTOW), in analogy to the concept of genetic tug-of-war for balancing other evolutionary constraints [34]. To facilitate mTOW’s genome-scale thermodynamic analysis, we implement a novel approach, Component Contribution Method (CCM) [35], for estimating reaction Gibbs energies. mTOW is applied to genome-scale metabolic network reconstructions of *E. coli* [36] and of *C. acetobutylicum* [37], and is shown to successfully explain measured metabolite concentrations in both species under various growth conditions [4,6].

## Results

### Computational estimation of metabolite concentrations via metabolic Tug-of-War (mTOW)

To identify the most likely metabolite concentrations for a given metabolic system, we present a novel computational approach, metabolic tug-of-war (mTOW), that searches for fluxes and metabolite concentrations, that (i) obey the second law of thermodynamics, (ii) whose total metabolite concentration (sum of all species) is limited, and (iii) whose enzyme levels are small, by ensuring that all active reactions are thermodynamically favorable with a reasonable driving force. The input data required for this method are the stoichiometry of the reactions in a given metabolic network (readily available for numerous microbes [38-43]) and reaction Gibbs energies.

The group contribution method (GCM) for estimating reaction Gibbs energies is a practical method for providing genome-wide coverage of metabolic reactions. The assumption that chemical groups are independent enables a state-of-the-art GCM approach to estimate the *ΔG*'^0^ of a major fraction of the reactions in a typical cell [44-46], but with a relatively high RMSE (root mean squared error) of 5.2 kJ/mol (according to a collection of 128 reactions from iAF1260 that have measured *ΔG*'^0^ data [47]). Here, we employ a novel method, Component Contribution Method (CCM) [35], which aims to improve GCM’s accuracy while keeping the same genome-wide scope. It is based on the fact that the standard Gibbs energy of 128 reactions in *E. coli*’s metabolism (including most reactions in glycolysis, pentose phosphate pathway, TCA cycle, etc.) have been directly measured and hence there is no need to reevaluate their energy based on group decomposition. CCM’s estimation of reactions Gibbs energy in *E. coli* shows a marked improvement in accuracy over GCM, with a RMSE of 2.9 kJ/mol (see further description of CCM and a comparison with GCM in the Methods and Supp. Material).

Absolute enzyme levels cannot be computed explicitly without extensive kinetic knowledge, including in vivo k_cat_ values, which are rarely available. However, there is a fundamental relationship between reaction thermodynamic driving force and the enzyme level required to maintain a given amount of net flux. For reactions far from equilibrium, changes to thermodynamic driving force have a negligible effect on the net flux. In contrast, for reactions close to equilibrium, the required enzyme level increases dramatically as the driving force approaches zero (i.e., where forward and reverse fluxes greatly exceed net flux) [48]. Equation 2 approximates the minimally enzyme level (*E*) required for maintaining a given net reaction rate (v) and the associated thermodynamic driving force (−*ΔG*'):

$$E=\frac{v}{w^{+}\left(1-e^{\frac{\Delta G'}{RT}}\right)}$$ (2)

The derivation of this formula is based on a decomposition of the reversible Michaelis-Menten rate laws into three terms representing (as in [48]) (i) enzyme levels, E, in units of [gr(enzyme)/gr(cell dry weight)], (ii) kinetic effect of substrate and product concentrations, denoted here by w+ (which is assumed to have a physiological bound to overcome lack of kinetic knowledge on enzyme k_cat_ and k_m_ values; see Supp. Material) and (iii) the effect of the thermodynamic driving force. Note that enzyme level rises and eventually becomes infinite as the thermodynamic driving force approaches zero (Figure S2 in File S1).

The complete formulation of mTOW involves a non-convex optimization, searching for a flux distribution and metabolite concentrations [19,49], minimizing both metabolite and enzyme levels (Methods). Since large-scale non-convex optimizations are computationally intractable, we obtain an approximated solution via a two-step optimization approach (Figure S3 in File S1): (i) solving a Mixed-Integer Linear Programming (MILP) problem to identify a thermodynamically feasible flux distribution under a growth medium at hand. To account for potential errors in the estimated Gibbs energy data obtained by CCM, mTOW minimizes the total sum of corrections to thermodynamic constants required to obtain a thermodynamically feasible flux distribution in this step. The model was further constrained by measured flux and growth rates on aerobic glucose-fed *E. coli* [5], and for *C. acetobutylicum* [6] (Supp. Material) (ii). This step involves solving a Quadratic Programming (QP) problem to compute optimal metabolite concentrations that satisfy the second law of thermodynamics, with minimal metabolite and enzyme levels (given the flux rates derived from the first step). Thermodynamic feasibility is enforced by requiring that *ΔG* for all forward reactions is negative (i.e. a positive thermodynamic force) [50]. To formulate this constraint as a linear equation, the optimization variables were defined as the log of metabolite concentrations (as done in [50]). Hence, the total sum of metabolite concentrations is approximated via a quadratic function, based on the sum of squared log-scale concentrations normalized by the minimal allowed concentration (Methods). Enzyme levels were also expressed via a quadratic function, based on the flux rate (v) and thermodynamic driving force of −*ΔG*' approximating Equation 2 (Methods). The tradeoff between the two optimization criteria is explored based on the concept of Pareto optimality (Methods). We show that the prediction performance of mTOW is robust to alternative possible flux distributions predicted in step (i) (Supp. Material).

Analyzing metabolite concentrations in both *E. coli* and *C. acetobutylicum*, we assume that metabolite concentrations are bounded between 10 nM (a concentration reflecting at least a single molecule per cell in these bacteria) and 100 mM (which is higher than the maximal measured concentration of any metabolite in these bacteria [4]). In both the *E. coli* and *C. acetobutylicum* models, extracellular metabolite concentrations were used to further constrain the solution space based on the specific growth media. A limited set of intracellular currency metabolites (ATP, ADP, AMP, NADH, NAD^+^ and Pi), were further constrained based on experimental measurements in each growth medium (see Supp. Material).

### Metabolite concentrations in *E. coli* across different carbon sources

**Figure 2:**
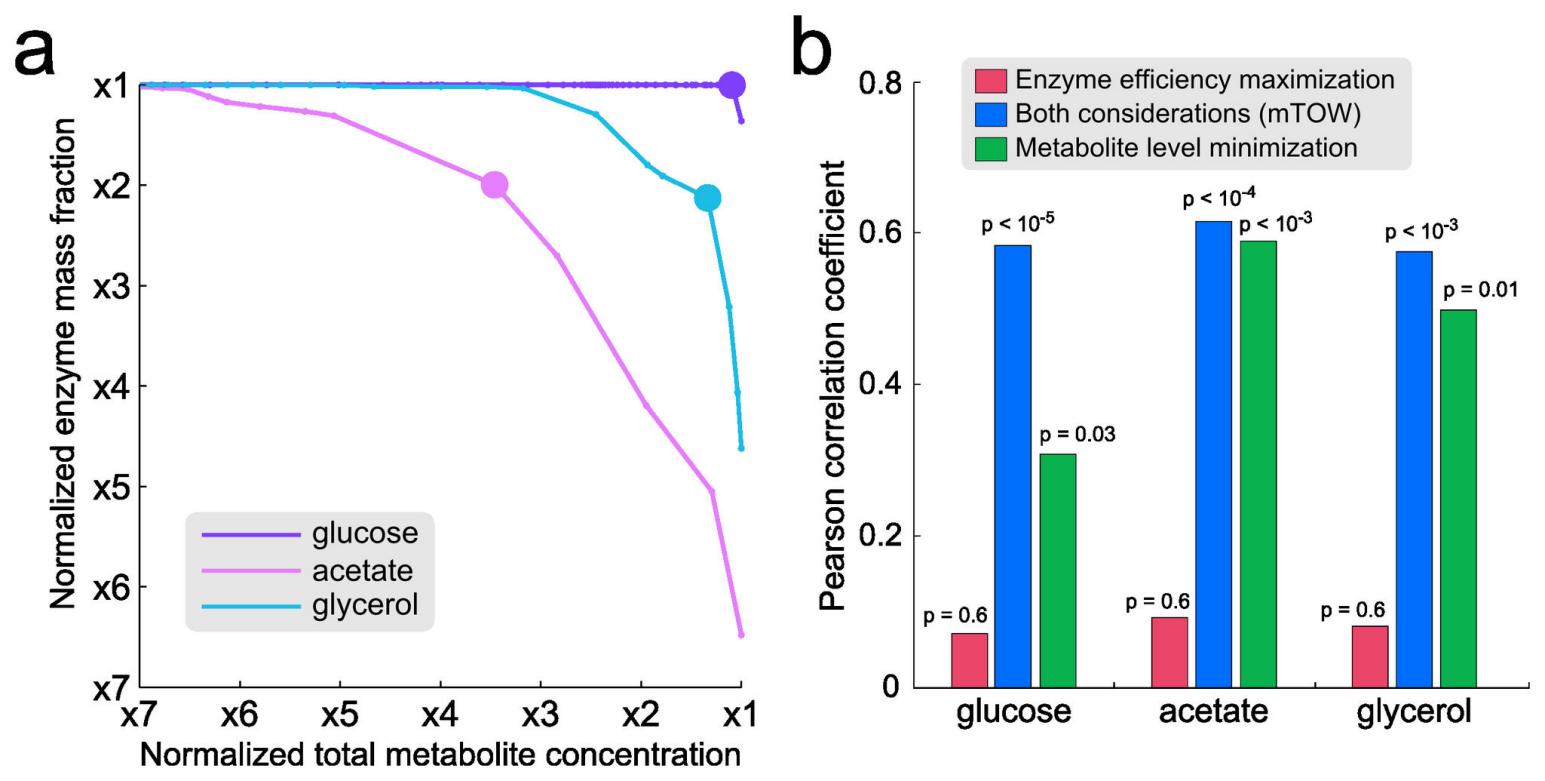
The contribution of the two optimization factors, minimization of metabolite load and maximization of enzyme efficiency, to the successful prediction of metabolite concentrations. (a) Pareto-optimal solutions predicted by mTOW for *E. coli* grown under glucose, acetate and glycerol media. Axes values are normalized to minimal values, where 1 represents the minimum, and the rest of the values represent the deviations (in fold change) from those minimal values. (b) The correlation between mTOW’s predicted and measured metabolite concentrations when considering either one of the optimization factors or both of them together.

We applied mTOW to predict metabolite concentrations in *E. coli* grown on glucose, acetate and glycerol media, for which high-throughput measurements are available for validation [4]. Using the genome-scale metabolic network model of *E. coli* by Feist et al. [36], we explored the tradeoff between the two optimality criteria. To this end, we employed the concept of Pareto optimality, which is useful in metabolic modeling when multiple optimization criteria are at hand [2]. Specifically, mTOW was applied to characterize a Pareto front, consisting of efficient solutions (i.e. metabolite concentration vectors) that cannot be improved with respect to one optimality criterion without damaging the other (Figure 2a). As shown, a clear trade-off between the two objectives exists for *E. coli* grown on acetate or glycerol, while a solution that is close to optimal in both objectives is achievable in glucose media. To choose a single biologically plausible mTOW solution per media (for validation against experimental data), we chose solutions achieving high values for both objectives (by considering the sum of the objective values, each normalized to its maximal attainable value). Using this strategy, mTOW predicts the concentration of 507, 412 and 412 metabolites in glucose, acetate and glycerol media, respectively (with the complete set of predictions provided in File S2).

These predicted concentrations corresponded well to the measurements of Bennett et al [4] (Table 1): Pearson correlation of log measured metabolite concentrations vs. log predicted concentrations in three carbon sources was, for glucose, R = 0.58 (p = 10-8; N = 57 metabolites); for glycerol, R = 0.57 (p = 0.002; N = 32 metabolites); and, for acetate, R = 0.54 (p = 0.004; N = 34 metabolites). Similar significant correlations were achieved when utilizing a Spearman correlation test (Table S2 in File S1). Notably, the overall prediction performance of mTOW remains highly robust to other choices of Pareto-optimal solutions. Specifically, examining the set of Pareto optimal solutions (per media) in which each of the objectives deviates in up to 20% from the chosen solution results in an average drop of only 5% in Pearson correlation with the measured metabolite concentrations (Table S3 in File S1). As a comparison, we checked whether metabolite concentrations could also be correctly predicted based on either the minimization of total metabolite level or maximization of enzyme efficiency alone. We found that utilizing only one of the criteria significantly compromises the predictions in all three growth conditions (Figure 2b). Overall, this result demonstrates the importance of jointly considering the dual physiological requirements when attempting to predict metabolite concentrations. In addition, when applying thermodynamics-based flux analysis (TMFA; a previous method that adds thermodynamic constraints to standard flux balance analysis) [19] to predict these measured concentrations, the correlations were found to be insignificant across all three growth media.

**Table 1:** Prediction performance for absolute metabolite concentrations in *E. coli* and *C. acetobutylicum* under various growth conditions achieved based on: (I) chemical properties [29], (II) mTOW, (III) mTOW when controlling for the variation in measured concentrations that is explained by the chemical properties fit (via Pearson partial correlation [58]), and (IV) via a regression model that combines both methods.

	***E. coli***				***C. acetobutylicum***	
**Pearson correlations (R) for log [predicted concentration] vs. log [measured concentration]**	**Aerobic Glucose**	**Aerobic Acetate**	**Aerobic Glycerol**	**Anaerobic glucose**	**Acido-genesis**	**Solvento-genesis**
**Chemical properties** [29]	0.57	0.41	0.47	0.14	0.16	0.12
	(p<10-9)	(p<10^-2^)	(p<10-3)	non-significant	non-significant	non-significant
**mTOW**	0.59	0.61	0.55	0.64	0.46	0.45
	(p<10-5)	(p<10-4)	(p<10-4)	(p<10-4)	(p<10-4)	(p<10-3)
**mTOW, controlling for chemical properties**	0.48	0.57	0.51	-	-	-
	(p<10-3)	(p<10^-2^)	(p<10^-2^)			
**Integrating mTOW and chemical properties prediction**	0.74	0.6	0.58	-	-	-
	(p<10-10)	(p<10-3)	(p<10-3)			

Chemical properties were unable to correctly predict metabolite concentrations in *E. coli* under anaerobic media and in *C. acetobutylicum*.

**Figure 3:**
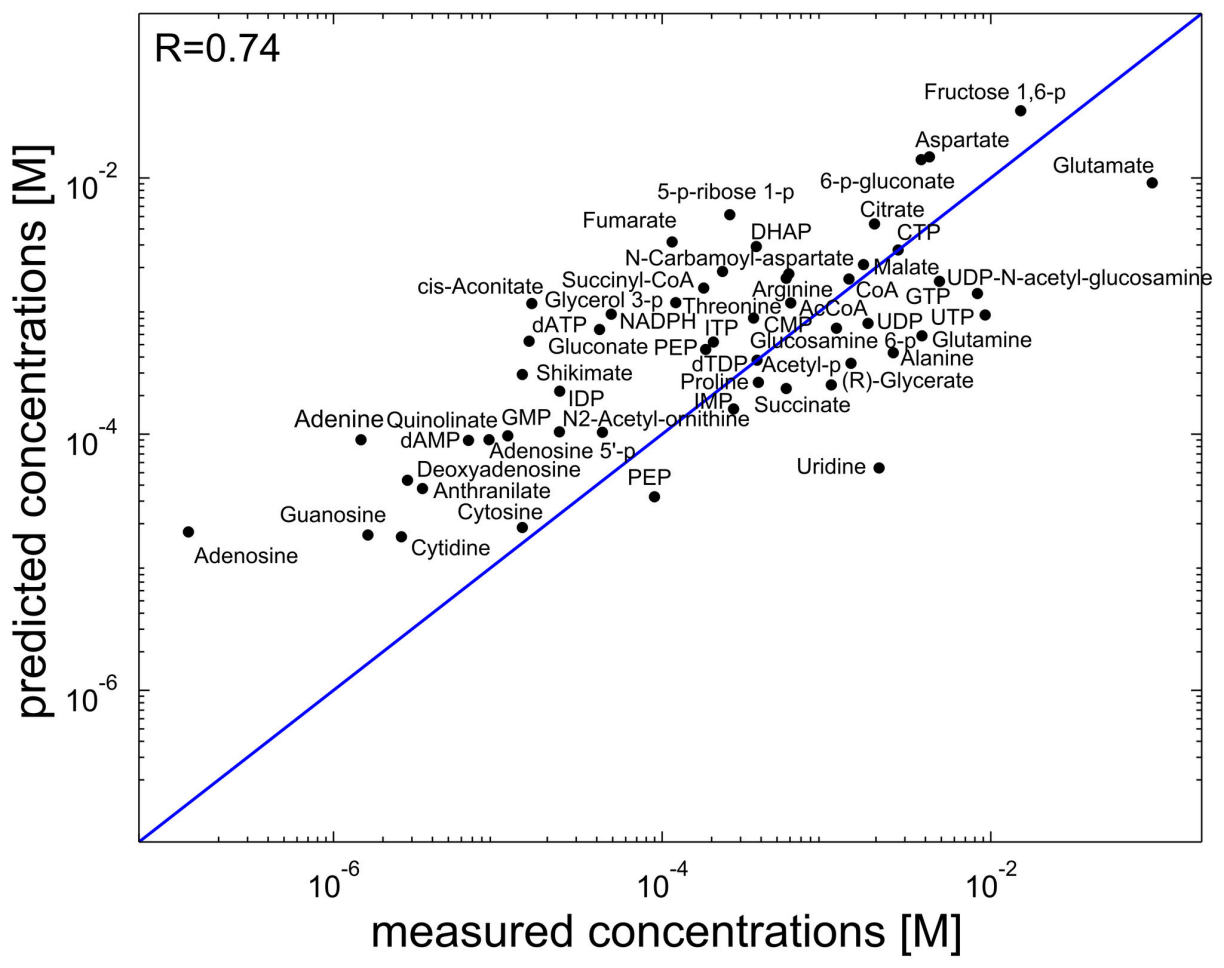
Measured metabolite concentration for aerobic glucose-fed *E. coli* versus predictions made by integrating mTOW with chemical properties-based concentration prediction.

As a further analysis, we applied a recently described method to predict metabolite concentrations based on chemical properties [29]. Specifically, metabolic concentrations were previously shown to positively correlate with metabolites’ non-polar surface area, and negatively correlate with number of charged atoms. We found that the correlation between the measured concentrations and predicted ones based on these chemical properties (using a simple linear regression model [29]) is comparable to that obtained by mTOW only under glucose media, but is markedly lower for glycerol and acetate media (and Table S2 in File S1). Next, we applied a Pearson partial correlation test to evaluate the correlation between mTOW’s predicted concentrations and the measured ones, while controlling for the variation in the measured concentrations that can also be explained by the chemical properties. We found a significant Pearson partial correlation under all three media, showing that the two prediction methods are complementary and explain different parts of the observed variation in metabolite concentrations (Table 1 and Table S1 in File S1). Finally, we integrated the predictions of both approaches using a linear regression model and obtained a marked improvement in overall prediction accuracy across in glucose and glycerol media, reaching Pearson correlations of 0.74, 0.6, and 0.58 for glucose, acetate and glycerol media, respectively (Table 1; Figure 3; Table S2 in File S1). To further quantify the difference between mTOW’s predictions and the measured dataset, we calculated a RMSE (root mean squared error) score. We find that mTOW’s predictions achieve a RMSE score of 0.8 orders of magnitude, which is comparable to the reported average experimental error of 0.5 orders of magnitude [4-6] (Table S4 in File S1).

**Figure 4:**
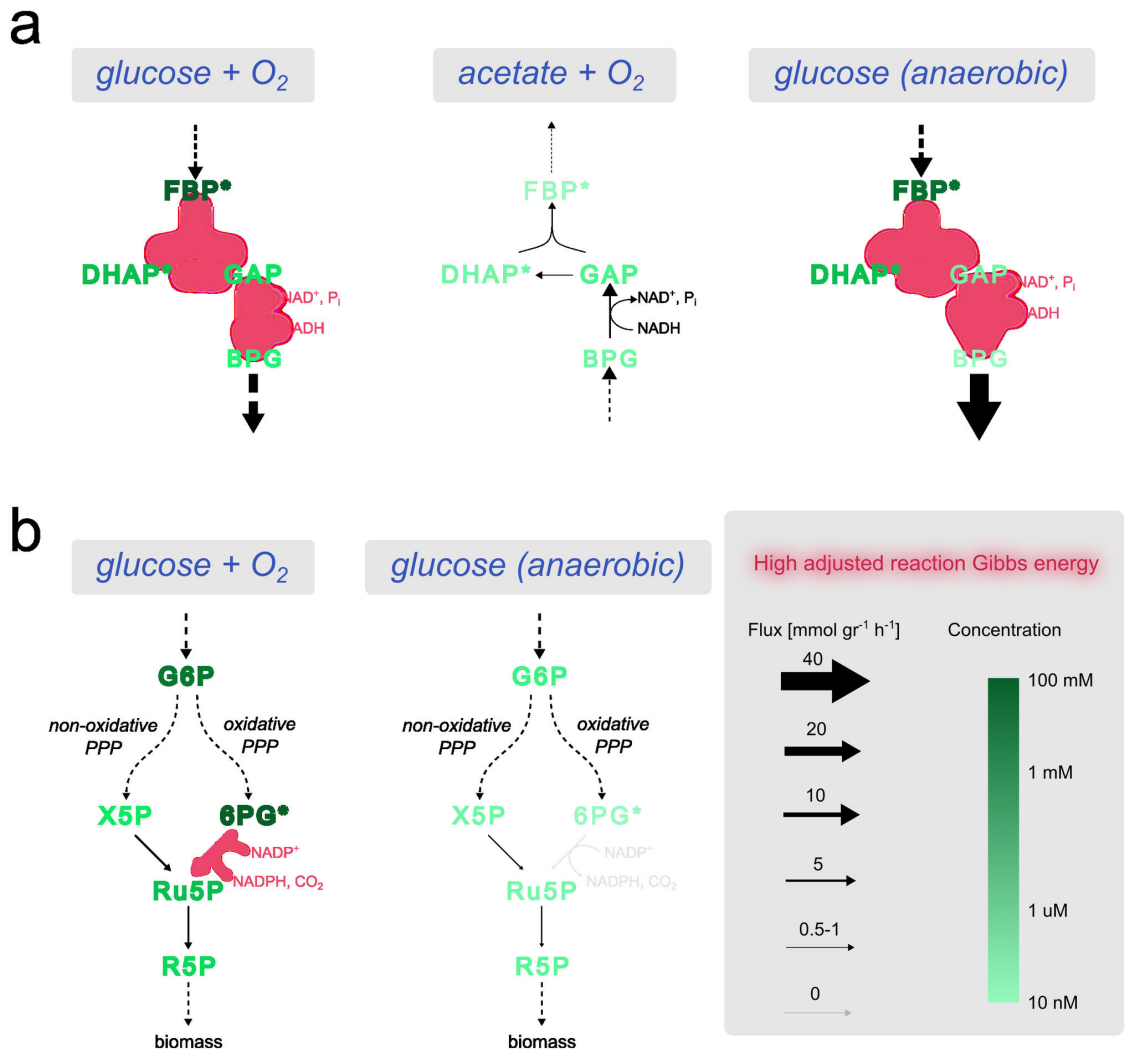
mTOW predictions of metabolite concentrations in glycolysis and pentose phosphate pathway are in accordance with experimental data. Reactions with high adjusted Gibbs energies (above 5.7 kJ/mol) are marked in red, and measured metabolites are marked with an asterisk. (a) On glucose media mTOW predicts a gradual decrease in metabolite concentrations across a distributed thermodynamic bottleneck from FBP to BPG (as supported by the measurements of FBP and DHAP) in both aerobic and anaerobic conditions. In aerobic acetate medium, the reversal of the glycolytic flux direction eliminates the thermodynamic bottleneck and leads to the prediction of markedly lower concentrations for FBP and DHAP in accordance with experimental data. (b) mTOW correctly predicts a marked decrease in concentration of 6PG in glucose media under anaerobic versus aerobic glucose conditions, due to thermodynamic considerations involving the decrease in flux through phosphogluconate dehydrogenase (that metabolite 6PG to Ru5P) in anaerobic conditions. Metabolite abbreviations presented in the figure: FBP-D-fructose 1,6-bisphosphate, DHAP - dihydroxyacetone phosphate, GAP-D-glyceraldehyde 3-phosphate, BPG-D-glycerate 1,3-bisphosphate, PPP - Pentose Phosphate Pathway, G6P - D-glucose 6-phosphate, X5P - D-xylulose 5-phosphate, 6PG - D-gluconate 6-phosphate, Ru5P - D-ribulose 5-phosphate, R5P - D-ribose 5-phosphate.

To gain further insight into mTOW’s metabolic concentration prediction, we analyzed in detail the predicted concentrations of glycolytic intermediate compounds, both in glucose and in acetate media, under aerobic conditions. During aerobic growth on glucose, glycolysis carries a high flux in the catabolic direction (from glucose to pyruvate), with the dual function of producing energy (ATP and NADH) and biomass precursors (e.g. amino acids; Figure 4a) [51]. To facilitate this high flux, the metabolite concentrations should overcome a distributed thermodynamic bottleneck (as defined in [52]; and in Supp. Material) consisting of three consecutive reactions with relatively high adjusted Gibbs energy (21 kJ/mol; when considering the measured concentrations of co-factors and the rest of the metabolites having a standard concentration of 1mM; Supp. Material): fructose-bisphosphate aldolase, triose-phosphate isomerase and glyceraldehyde-3-phosphate dehydrogenase. In this case, mTOW predicts a gradient of decreasing concentrations (as in the example on Figure 1b) from the initial substrate fructose-1,6P and up to glycerate-1,3P, in agreement with experimental measurements (Figure 4a). The predicted concentration drop per each of these reactions provides high enough thermodynamic driving force (>3 kJ/mol per reaction). The transition of *E. coli* from glucose as a carbon source to acetate requires the reversal of glycolytic flux and the activation of gluconeogenesis [2,53]. The overall flux rate through gluconeogenesis is significantly lower than that of glycolysis since it is used only for biomass and not energy production [31]. These flux changes in acetate media eliminate the mentioned thermodynamic bottleneck (with all three reactions operating in their thermodynamically favorable direction). This leads to a prediction of a markedly lower concentration of fructose-1,6P and dihydroxyacetone-P in acetate versus glucose media, in agreement with experimental measurements (Figure 4b) [31].

### Metabolite concentrations in aerobic and anaerobic conditions for *E. coli*

We have demonstrated above that mTOW predicts metabolite concentrations in *E. coli* under various aerobic growth conditions. Next, we investigated its performance in predicting absolute concentrations under anaerobic growth conditions. To obtain data on absolute concentrations of metabolites in *E. coli* under anaerobic conditions, we applied an established LC/MS based metabolomics approach as described in the methods section [4]. This experiment quantified the absolute concentrations of 56 metabolites (see measurements in Supp. Material). These were compared to mTOW’s predictions for the same metabolites in anaerobic glucose minimal media. The measured and predicted concentrations correlated well (Pearson R = 0.64; p = 10-4; Table 1). Comparing also the changes in concentration between aerobic and anaerobic conditions resulted in a significant Pearson correlation of 0.44 (p-value = 0.02).

The metabolite showing the largest change in measured concentration between aerobic and anaerobic conditions is gluconate-6P, whose anaerobic concentration is 6-fold lower. mTOW correctly predicts that gluconate-6P has the largest drop in concentration under an anaerobic medium due to thermodynamic considerations involving the change of flux through the oxidative and the non-oxidative branches of the pentose phosphate (PP) pathway (Figure 4b). Specifically, in aerobic conditions, ribose-5P is produced predominantly via the oxidative PP pathway, which ends in the decarboxylation of gluconate-6P to ribulose-5P (catalyzed by phosphogluconate dehydrogenase). This reaction has an adjusted Gibbs energy of 8 kJ/mol, and thus requires a high gluconate-6P concentration to maintain a reasonable thermodynamic driving force. In anaerobic conditions, mTOW predicts a drop in flux through the oxidative branch of the PP pathway (involving lower flux through phosphogluconate dehydrogenase), which is compensated by an increased flux through the non-oxidative branch of PP to synthesize ribose-5P. To validate mTOW’s prediction, we measured the ratio of ribose-5P synthesized via the oxidative versus non-oxidative branches of the PP pathway (by measuring the steady-state labeling of ribose-5P when feeding *E. coli* with 1,2-13C-glucose in aerobic and anaerobic media; Methods). Our measurements confirm mTOW’s prediction, showing a marked decrease of ~33% in flux through the oxidative branch of PP pathway in anaerobic conditions (Figure S6 in File S1).

Observing glycolysis, the predicted metabolite concentrations are mainly affected by the higher glycolytic flux [2,53] and the lower oxidative state (NAD/NADH ratio) under anaerobic vs. aerobic growth. This contributes to an even larger predicted concentration gradient across metabolites under anaerobic conditions with a decrease of two orders of magnitude in the concentration of the downstream metabolite in this system, glycerate-1,3P.

### Analyzing metabolite concentrations in *C. acetobutylicum* during acidogenesis and solventogenesis growth phases

To evaluate mTOW’s ability to predict metabolite concentrations in another microorganism whose overall metabolism is less well characterized than that of *E. coli*, we examined the biofuel producer *C. acetobutylicum*. We analyzed metabolite concentrations in this organism during its acidogenic growth phase (characterized by exponential growth and high rates of acid secretion) and its solventogenic growth phase (stationary phase with high secretion rates of solvents such as acetone and butanol), using a metabolic network model by Lee et al [37].

Applying mTOW to predict metabolite concentrations under both growth phases resulted in the prediction of 206 and 217 concentrations for acidogenesis and solventogenesis, respectively. Comparing the predicted concentrations with 40 measured concentrations collected by Amador-Noguez et al [6], mTOW obtains (on a log scale) a significant Pearson correlation of R = 0.46 (p = 8·10-4) and R = 0.45 (p = 10-3) for acidogenesis and solventogenesis, respectively (Table 1; File S1). Close inspection of specific metabolites revealed that in both phases, fructose 1,6P and aspartate were predicted to have the highest allowed concentration, in agreement with their high measured concentrations (above 1mM). In addition, glutamine had high predicted and measured concentrations in acidogenesis, and glycerate-3P in solventogenesis, whereas both citrate and malonyl-CoA were predicted to have low concentrations (< 10-3 mM) in both conditions.

## Discussion

We demonstrated that under some growth media metabolite concentrations reflect a compromise between cellular adaptations towards minimizing the total metabolite vs. enzyme levels. Since metabolite and enzyme levels cannot be explicitly computed based on metabolic fluxes without extensive kinetic knowledge, we approximated enzyme levels for reactions based on their thermodynamic forces, as low thermodynamic forces result in substantial backward fluxes, and thus higher required enzyme levels. mTOW does not take into account how enzyme kinetic considerations influence metabolite concentrations, for example by changing an enzyme rate via increasing both its substrate and product concentrations proportionally (i.e. without changing its thermodynamic driving force). Furthermore, it does not account for allosteric regulation or for the effect of specific enzyme kinetic parameters on the enzyme levels required to catalyze a unit of flux. mTOW’s predictions also implicitly assume that enzymes are fully saturated, an assumption which is partially supported by experimental evidence [4]. While mTOW’s estimated enzyme levels should be regarded as a very crude approximation, we evaluate the plausibility of enzyme predicted concentrations under glucose medium with the proteomic data of Taniguchi et al [54] and Ishii et al [5]. We find a statistically significant correlation of 0.32 (p = 10-6 for 210 enzymes) between the predicted and measured enzyme levels using the recent proteomics data of Taniguchi et al, and a correlation of 0.44 (p = 0.02 for 26 enzymes) using the data of Ishii et al. (File S1).

To enable the prediction of metabolite concentrations via a genome-scale flux and thermodynamic analysis, mTOW is bound to make additional simplifying assumptions that may bias its predictions: First, it relies on estimated Gibbs energies for reactions via the CCM method rather than solely on experimental data which is lacking for most of the reactions in *E. coli*. Second, although the coverage of group contribution-based methods for estimating thermodynamic parameters (such as CCM) is much higher than what can be derived from experimental data, it is still not complete and covers an average of 90% of the active (i.e. flux carrying) reactions across the various growth media (and 75% of the entire reactions set in *E. coli*). Third, the exact intracellular conditions (which include ionic strength, pH, and pMg) affect the energetics of reactions [55], and current thermodynamic models are lacking in their ability to model these changes precisely. Though CCM corrects for pH and ionic strength, accurate prediction of such changes across media and their effect on metabolism remains an open challenge. Fourth, current metabolic models do not encompass all relevant known reactions. Specifically, while the model accounts for the required biosynthetic rate of many essential biomass precursors, it does not include information on the thermodynamic requirements of the reactions that consume them as these are outside the scope of the model. For example, the employed *E. coli* model lacks information on the thermodynamics of aminoacyl tRNA synthetase that utilizes amino-acids, which is likely to limit mTOW’s ability to correctly predict amino-acid concentrations (and potentially that of their upstream intermediates; see Supp. Material). Fifth, as mTOW is formulated as a non-convex optimization problem, whose exact solution is computationally intractable for large-scale networks, we obtain only approximated solutions based on a combination of mixed-integer linear programing and quadratic programming optimizations (see Supp. Material).

Identifying optimization criteria that would explain complex metabolic behaviors remains a major open challenge in systems biology [56]. The fact that mTOW successfully predicts metabolite concentrations based on a compromise between two objectives and without requiring a detailed kinetic model should provide further support for this line of research.

## Methods

### Estimating reaction Gibbs Energies via Component Contribution Method (CCM)

**Figure 5:**
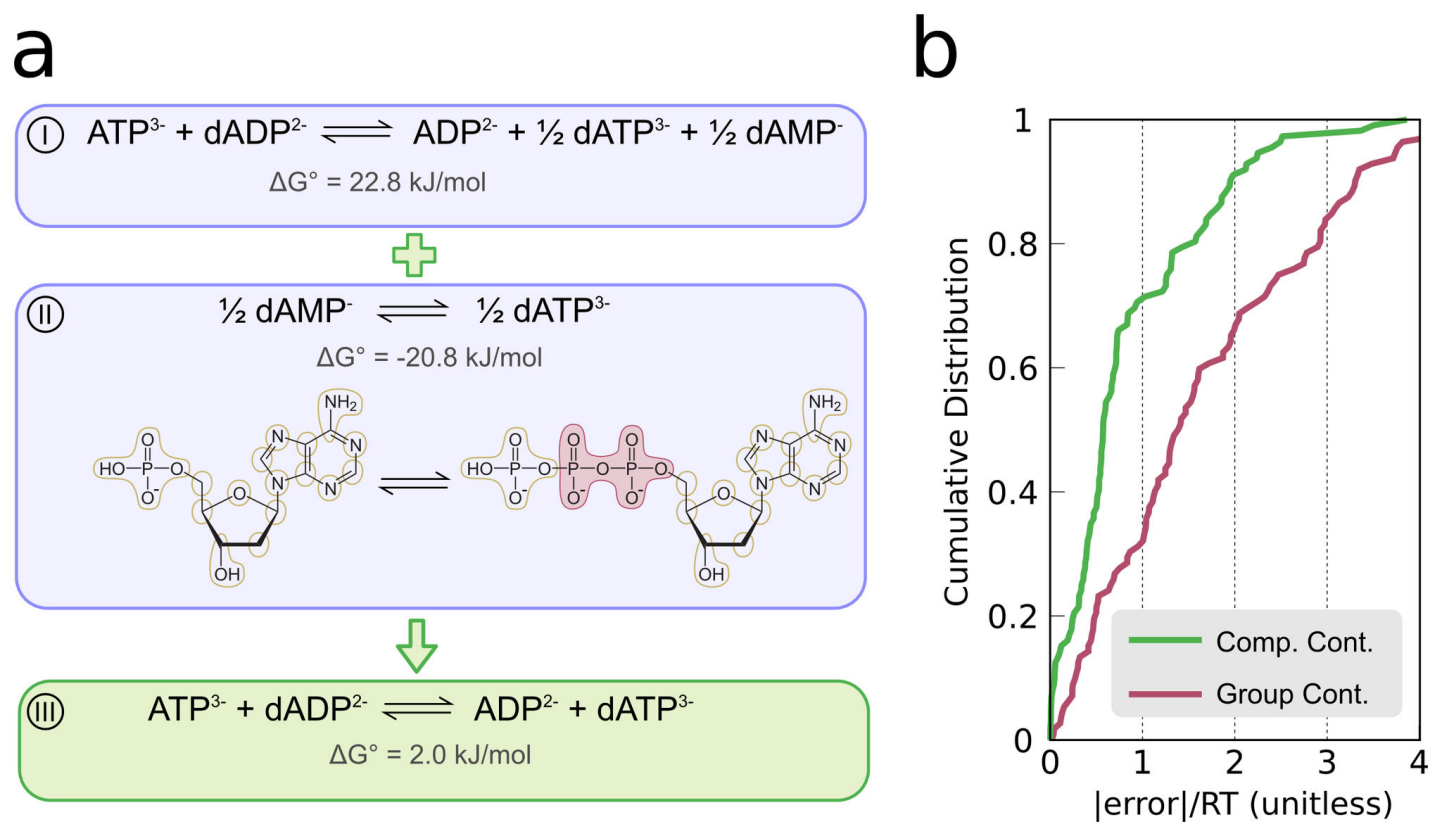
Using CCM to evaluate a reaction Gibbs energy. (a) The reaction catalyzed by phosphoribosyl formyl glycinamidine synthase (I) involves mostly compounds from TECRDB and two others: N2-Formyl-N1-(5-phospho-D-ribosyl) glycinamide (FGAM) and 2-(Formamido)-N1-(5-phospho-D-ribosyl) acetamidine (FPRAM). In this case, CCM divides the reaction into two parts: one which can be completely evaluated using directly observed reaction data (II) and another which can only be resolved using group contributions (III). The five groups that change throughout this reaction are highlighted in red. The final *ΔG*'^0^ estimation is simply the sum of these two half-reactions: -164.0 + 204.9 = 40.9 kJ/mol. The last stage in the algorithm is to apply the Legendre transform [59] (using pKa data from ChemAxon; see Supp. Material). The result in this case is *ΔG*'^0^= -39.8 kJ/mol. The previous GCM prediction for the same reaction is *ΔG*'^0^= -90.8 kJ/mol (as appears in iAF1260). Note that the values appearing in iAF1260 are also Legendre transformed. (b) The cumulative distribution functions of the absolute errors for CCM and versus that of GCM taken from the iAF1260 model. The error was calculated by comparing the prediction to the median value for that reaction taken from TECRDB. Only reactions that appear in all three datasets are shown (113 in total). The intersections with the dashed lines indicate the fraction of reactions whose predicted value are in the range of ±RT (2.5 kJ/mol) of the value in TECRDB. One can see that for CCM, 70% of these reactions are in this category, compared to only about 30% in iAF1260.

Given a set of metabolic reactions, CCM assigns them to the following categories:

i. Reactions that can be completely determined using a linear combination of measured reactions (from the TECRDB [47]).
ii. Reactions for which group contributions must be used for all reactants in order to estimate the *ΔG*'^0^ (same as in standard GCM).
iii. Reactions that can be decomposed to two half-reactions, one which is in category I and the other in category II (e.g. the reaction in Figure 5a).

The assignment of reactions to these categories is optimal in terms of preferring assignments to the first and then third categories (in accordance with the expected higher accuracy of the Gibbs energy estimations in the first category). The resulting set of Gibbs energy estimations for the reactions in all three categories combined is guaranteed to be consistent with the first law of thermodynamics (which in this case means that all reaction Gibbs energies can be described in terms of changes in the metabolites’ Gibbs energy of formation).

Comparing the distribution of errors in the Gibbs energy estimations made by CCM to that obtained by standard GCM (for 128 reactions with measured data in *E. coli*), shows a marked advantage to CCM (Methods; Figure S1 in File S1; File S3). Specifically, in CCM, about 70% of these reactions have an error smaller than RT (2.5 kJ/mol) compared to only 30% for GCM (Figure 5b). The deviations in CCM are attributed to two factors: (i) measurement errors that cause the set of reaction Gibbs energies to be inconsistent (i.e. violate the first law of thermodynamics), (ii) errors in the process of normalizing the effect pseudoisomers (either due to errors in the pH and ionic strength annotations or in the pKa values used for each compound) [46]. Notably, these errors affect the standard GCM methods as well, and are added on top of the inherent error caused by the underlying assumption that group contributions are independent of each other.

Importantly, although these 128 measured reactions comprise less than 10% of the reactions in the iAF1260 model, they cover approximately 70% of the total absolute flux in mTOW’s solutions (across all media). Moreover, there are about 400 other reactions that fall in category I, and their CCM estimations are expected to be more accurate than the GCM-based ones, since the estimation does not use the simplifying assumption needed for the group contribution method. To support this claim, we ran a cross-validation test based on the leave-one-out method and found that, when using CCM instead of standard GCM, there is a 42% decrease in the median error for reactions in category I, and a 20% decrease on average in all categories.

### mTOW’s formulation

mTOW aims to find a flux distribution (v) and metabolite levels (c) with low enzyme cost and metabolite load and can be formulated as:

$$\begin{array}{c} \min\;\sum_{i=1..m}M\left(c_{i}\right)+\delta \cdot \sum_{j\in R_{G}}E\left(c,v_{j}\right)\\ s.t.\\ \begin{array}{cccc} & S\cdot v=0 & & \left(1\right)\\ & v\geq 0 & & \left(2\right)\\ & \ln\left(c^{L}\right)\leq \ln\left(c\right)\leq \ln\left(c^{U}\right) & & \left(3\right)\\ & -\frac{\Delta G{'}_{j}^{0}}{RT}-S{'}_{j}\cdot \ln\left(c\right)\geq \beta & v_{j}>0\wedge j\in R_{G} & \left(4\right) \end{array} \end{array}$$

where Equation 1 represents stoichiometric mass-balance constraint, with S representing an nxm stoichiometric matrix. Equation 2 requires all reactions to have a non-negative flux rate (requiring that the reversible reactions be split into two irreversible ones prior to solving this optimization problem). Equation 3 restricts metabolite concentrations to a pre-defined range (between cL = 10-5 mM and cU = 100 mM). Equation 4 enforces the second law of thermodynamics, with *S*'_*j*_⋅*ln*(*c*) representing the inner product of the jth column in the stoichiometric matrix with the ln of metabolite concentrations. For reactions for which standard Gibbs energy data is available (whose set is denoted by RG), the thermodynamic driving force is computed based on the metabolite concentrations, with R and T denoting the gas constant (kJ/mol*K and temperature (K), respectively, and $\Delta G{'}_{j}^{0}$ represents the standard Gibbs energy for reaction j. Equation 4 can be transformed to a linear form via the usage of integer variables (as described [12,13]; Supp. Material). *β* represent a minimal value from which a reaction can operate (see below). The first term of the optimization function represents the minimization of total metabolite concentrations, while the second term represents the minimization of total enzyme concentrations. Hence, ideally, M(c), should be equal to c, and E(c,vj) should be equal to$\frac{v_{j}}{v^{+}\left(1-e^{\frac{\Delta G{'}_{j}}{RT}}\right)}$. For w+ we assumed a characteristic value of w+=1000 mmol/(gr*h), based on median enzyme kinetic data from BRENDA [57] (see Supp. Material). *δ* represent the weighting between the two optimization criteria, while varying it between 0 and inf enables to obtain a set of Pareto optimal solutions.

As described above, we obtained approximated solutions to this non-convex optimization by solving a MILP problem to first find a feasible flux distribution (v), followed by a QP problem to identify metabolite concentrations (c) with minimal metabolite and enzyme concentrations (see details in Supp. Material). In the QP problem, the total sum of metabolite concentrations is approximated by:

$$\tilde{M}\left(c_{i}\right)={\left(\ln\left(\frac{c_{i}}{c^{L}}\right)\right)}^{2}$$ (3)

where ci is the predicted concentration for metabolite i, and cL is the minimal concentration value (set to 10 nM).

Similarly, the enzyme level terms, $\tilde{E}\left(c,v_{j}\right)$, were approximated based on the catalyzed flux rate (v) and thermodynamic driving force of−*ΔG*'_*j*_, as follows:

$$\tilde{E}\left(c,v_{j}\right)=\{\begin{array}{cc} \infty & -\frac{\Delta G{'}_{j}}{RT}<\beta \\ v_{j}\cdot {\left(\alpha +\frac{\Delta G{'}_{j}}{RT}\right)}^{2} & \beta <-\frac{\Delta G{'}_{j}}{RT}<\alpha \\ 0 & \alpha <-\frac{\Delta G{'}_{j}}{RT} \end{array}$$ (4)

where α represents the thermodynamic driving force above which any increase would have a negligible effect on the enzyme level required to catalyze a unit of flux. Further details regarding the enzyme approximation, its units, and computation of these thresholds are given in the Supp. Material. Here, we describe the results obtained with *α*=4 (corresponding to a *ΔG*' of -10 kJ/mol) and *β*=0.02 (corresponding to a *ΔG*' of -0.05 kJ/mol) while as discussed in the Supp. Material, the results are robust for a wide range of parameter choices (Figures S4-S5 in File S1).

### Media and culture conditions and metabolite extraction

Wild-type K-12 strain NCM3722 of *E. coli* was cultured in minimal medium containing 4.7g/L KH2PO4, 13.5g/L K2HPO4, 1 g/L K2SO4, 0.1g/L MgSO4·7H2O, 10 mM NH4Cl, and 4g/L glucose. Experiments used for the determination of intracellular metabolite concentrations were performed using filter cultures. Agarose plates for filter cultures were prepared by mixing agarose with the above media composition to a final concentration of 1.5% agarose. To prepare filter cultures, a single colony was picked from a Luria Broth plate, and grown to saturation overnight in minimal media. The saturated overnight culture was then diluted to OD650 (optical density at 650 nm) of 0.03 into liquid minimal medium. This liquid culture was grown to OD650 of ~ 0.1, and transferred to a filter culture as follows: for each filter culture, 1.6 mL of liquid culture was passed through a 47 mm diameter round nylon filter (Millipore) and the filter placed cell-side up onto a medium-loaded agarose plate. The filter cultures were allowed to grow to an OD650 of 0.35 at which point metabolism was quenched and cells extracted by dropping the filters directly into 1.6 mL of −20°C 40:40:20 acetonitrile: methanol: water. For the growth of anaerobic cultures all procedures were carried out inside an anaerobic chamber (Bactron IV SHEL LAB) with an atmosphere of 90% nitrogen, 5% hydrogen, and 5% carbon dioxide.

Steady state labeling experiments using 1,2-13C-glucose were performed in liquid cultures under aerobic and anaerobic conditions. To prepare liquid cultures, a single colony was picked from a Luria Broth plate, and grown to saturation overnight in minimal media containing 1,2-13C-glucose (4g/L). The saturated overnight culture was then diluted to OD650 of 0.03 into liquid minimal medium containing 1,2-13C-glucose (4g/L). This liquid culture was grown to OD650 of ~ 0.3. 5ml of this liquid culture was passed through a 47 mm diameter round nylon filter (Millipore) and the filter was dropped immediately into 1.6 mL of −20°C 40:40:20 acetonitrile: methanol: water.

### Metabolite measurements

Cell extracts were analyzed by reversed-phase, ion-pairing liquid chromatography coupled by electrospray ionization (ESI; negative mode) to a high-resolution, high-mass-accuracy mass spectrometer (Exactive; Thermo, Fisher) operated in full scan mode for the detection of targeted compounds based on their accurate masses. This analysis was complemented with liquid chromatography coupled by ESI (positive and negative mode) to Thermo TSQ Quantum triple quadrupole mass spectrometers operating in selected reaction monitoring mode. Measurements from aerobic and anaerobic cell extracts were performed in parallel during the same day. Concentrations were determined based on the ratio of metabolite peak heights from aerobic and anaerobic cultures and using previously published metabolite concentrations from aerobic *E. coli* filter cultures grown under identical conditions [4]. The steady-state labeling patters of ribose-5-phosphate obtained when cells are grown in 1,2-13C glucose (100%) were used to calculate the fraction of ribose-5P produced via the oxidative PP pathway during aerobic and anaerobic conditions (Figure S6 in File S1).

## Supporting Information

File S1 **mTOW supplementary.** Supplementary file also containing all supplementary tables and figures.
(DOCX)

File S2 **mTOW predictions.**
(XLSX)

File S3 **Gibbs energy estimations made by CCM.**
(XLSX)
