# Supplementary material for: Steady-State Metabolite Concentrations Reflect a Balance between Maximizing Enzyme Efficiency and Minimizing Total Metabolite Load
Source: PLoS One. 2013 Sep 26;8(9):e75370. doi: 10.1371/journal.pone.0075370 (PMC3784570; doi:10.1371/journal.pone.0075370)
Supplement: File S1 — mTOW supplementary. Supplementary file also containing all supplementary tables and figures. (DOCX) [file pone.0075370.s001.docx]

Steady-state metabolite concentrations reflect a balance between maximizing enzyme efficiency and minimizing total metabolite load

Naama Tepper^1+^, Elad Noor^2+^, Daniel Amador-Noguez^3^, Hulda S. Haraldsdóttir^4^, Ron Milo^2^, Josh Rabinowitz^3^, Wolfram Liebermeister^5­­^ & Tomer Shlomi^1^*

^1^Dept. of Computer Science, Technion–IIT, Haifa 32000, Israel
^2^Department of Plant Sciences, Weizmann Institute of Science, Rehovot 76100, Israel
^3^Chemistry and Integrative Genomics, Princeton University, Princeton, NJ  08544
^4^Center for Systems Biology,University of Iceland, Sturlugata 8, 101 Reykjavik, Iceland
^5^Institut fürBiochemie, Charite -Universitätsmedizin Berlin, Berlin, Germany
+ equal contribution

*To whom correspondence should be addressed. E-mail: [tomersh@cs.technion.ac.il](mailto:tomersh@cs.technion.ac.il)

Contents

[1. 1. Estimating reaction Gibbs Energies via Component Contribution Method (CCM) 2](#_Toc333905086)

[1.1 Evaluation of the CCM method 3](#_Toc333905087)

[2. Effect of thermodynamic driving force on protein burden 3](#_Toc333905088)

[2.1 Effective cost of small thermodynamic forces 3](#_Toc333905089)

[2.2 Quantitative estimates 5](#_Toc333905090)

[2.3 Penalty term for small thermodynamic forces 5](#_Toc333905091)

[2.4 Derivation of the formulae 6](#_Toc333905092)

[3. Implementation of mTOW as two sub problems 7](#_Toc333905093)

[3.1 mTOW Step I: Predicting a thermodynamically feasible flux distribution 7](#_Toc333905094)

[3.2 mTOW Step II: Predicting metabolite concentrations 8](#_Toc333905095)

[3.3 Robustness analysis of mTOW’s implementation to the specific choice of flux distribution 9](#_Toc333905096)

[4. Distributed Thermodynamic Bottlenecks forces a concentration gradient 10](#_Toc333905097)

[5. The metabolic scope of mTOW predictions 11](#_Toc333905098)

[6. References 12](#_Toc333905099)

[7. Supplementary Figures 13](#_Toc333905100)

[8. Supplementary Tables 20](#_Toc333905101)

## Estimating reaction Gibbs Energies via Component Contribution Method (CCM)

CCM is based on a hierarchical linear regression technique which can be used to derive reaction Gibbs energies for genome-wide models. The first stage is to linearize the problem of converting formation energies to reaction energies by applying the reverse Legendre transform on the observed equilibrium constants in TECRDB (same as in [[1](#_ENREF_1)]). We start by defining $S$ as the stoichiometric matrix of measured reactions and a vector of measured reaction Gibbs energies $\Delta_{r}G’^{\circ}$. The linear nature of Gibbs energy means that for any linear combination of reaction stoichiometries (columns in the matrix $S$) the Gibbs energy can be calculated by applying the same linear transformation on the measured Gibbs energies. Therefore, the subspace spanned by the columns of $S$ represents the subspace of reactions which can be evaluated directly without using group contributions.

From the first law of thermodynamics, the change in Gibbs energy for a null-reaction (a column vector with only zeros) must be always 0. This means that any vector ($v$) in the nullspace of $S$ (i.e. $v$ satisfies $Sv = 0$), must satisfy $\Delta_{r}G’^{\circ}\cdot v=0$. In other words, $\Delta_{r}G’^{\circ}$ must be orthogonal to $\mathrm{null}(S)$. From the fundamental theorem of linear algebra we know that the nullspace is the orthogonal complement of the row space, therefore $\Delta_{r}G’^{\circ}$ must be in the row space of $S$. In practice, this is not true since $\Delta_{r}G’^{\circ}$ are empirically derived and thus subject to measurement noise. Also, the exact ionic strength is not known for most measurements and the theory of thermodynamics in aqueous solutions (which the reverse Legendre transform is based on) is itself an approximation that could deviate from reality. We thus project the vector $\Delta_{r}G’^{\circ}$ on the row-space of$S$ using an orthogonal projection in order to make it consistent with our assumptions.

After making $\Delta_{r}G’^{\circ}$ consistent with the first law of thermodynamics by projecting it onto the row-space of $S$, we can use the adjusted values, denoted by $\tilde{\Delta_{r} G’^{\circ}}$, to calculate the reaction Gibbs energy of every reaction in the column-space of $S$. Explicitly, given a reaction $r$ in the column-space of $S$, there is a vector $v$ which satisfies $Sv = r$. The Gibbs energy for this reaction would thus be equal to $\tilde{\Delta_{r} G’^{\circ}}\cdot v$.

The column-space of S represents only a fraction of the entire space of reactions, and thus most reactions are underdetermined by the linear system, e.g. any reaction that involves compounds that do not appear in TECRDB. For all such reactions, the group contributions are used to fill the gap of missing formation energies. In practice, most metabolic reactions in the cell are a combination of these two cases – a part that includes a combination of reactants for which the reaction energy can be directly estimated from a combination of measured reaction energies and a part that can be estimated indirectly based on the the change in groups and estimation of each group contribution. Mathematically this means the reaction is composed of two orthogonal components: one within the column-space of $S$ and the other in the nullspace of $S$. Thus, the final estimation for the Δ_r_G’^0^ of such a reaction is the sum of both estimations, where each component is computed independently.

### Evaluation of the CCM method

Some of the reactions in iAF1260 have measured values in the NIST database of thermodynamic empirical data of enzyme catalyzed reactions (TECRDB). We have identified 128 such reactions which have a measured equilibrium constant in the pH range of 6-8. For each such reaction, we calculate the average $\Delta G^{'\circ}$ over these measurements and compare that to the estimations in the iAF1260 model and according to CCM.

Overall, we find that the new estimations from CCM fit the measured data better (RMSE = 2.9 kJ/mol) than the previous estimations found in iAF1260 [[2](#_ENREF_2)] (RMSE = 5.2 kJ/mol) as shown in Supp. Figure S1. This can be attributed to the fact that the latter are derived from standard GCM which uses the group contributions to estimate the reaction Gibbs energy even when there is explicit data for that reaction in the training set.

To further evaluate the improvement of CCM compared to standard GCM, we performed a cross-validation test for the measured data used in the training set. Since the specific implementation of GCM used for deriving the $\Delta G^{'\circ}$ in the iAF1260 model was not available to us, we used our own implementation for this cross-validation. We then applied the leave-one-out methodology on every single reaction in our training database and compared the predictions of CCM and GCM to the measured value. We then calculated the median error for (i) all reactions in the training set and (ii) only those reactions which are linearly dependent on the other reactions in the training set. The second set of reaction represents reactions which do not require any group decomposition in order to evaluate their Gibbs energy, and thus CCM is expected to have a greater advantage for them.

The results for this cross-validation test are: (i) an overall reduction of 20% in the median error for all 633 estimated reactions, and (ii) a 42% reduction in the median error for the 326 linearly dependent reactions.

## Effect of thermodynamic driving force on protein burden

Chemical reactions are driven by thermodynamic forces. Rates and forces depend on the reactant concentrations, but there is no fixed relationship between them. Nevertheless, rates tend to rise with the thermodynamic force, and if cells have to maintain certain metabolic fluxes, small thermodynamic forces need to be compensated by higher enzyme levels. Therefore, insufficient forces effectively entail enzyme costs. Here we estimate this cost based on simplifying assumptions. We compare the predicted enzyme levels to known protein abundances and derive a simple penalty term for small thermodynamic forces in constraint-based models.

### Effective cost of small thermodynamic forces

In thermodynamic flux analysis, the directions of metabolic fluxes are related to the thermodynamic forces $-\Delta G$: a flux can only be positive if there is also a positive force. In our method for concentration predictions, we apply an even stricter constraint: to allow for a positive flux, the force has to exceed some positive minimal value $\beta$, and forces between this value and a higher threshold $\alpha$ are penalized by a quadratic penalty term. We chose a quadratic penalty as an estimator to the real cost discussed below in order to enable efficient solution using quadratic linear programming. Thus, forces should not be too small. How can we justify this assumption? Here we argue that cells that need to sustain a certain predefined flux would have to compensate small thermodynamic forces by higher enzyme levels. Small forces will translate into a higher enzyme burden; this is the meaning of the penalty term.

The main assumption in this argument is that reaction rates, at a fixed enzyme level, tend to increase with the force. We shall show this for reversible rate laws of the form *v =* $E(w^{+}-w^{-})$ where *E* is the enzyme level, $w^{+}$ is the microscopic rate per gram of enzyme in the forward direction and $w^{-}$ is the same just in the reverse direction. The units typically used for $w^{\pm}$ are $mmol gr\left( enz. \right)^{-1}h^{-1}$. As shown in [[3](#_ENREF_3)] and below, the ratio of microscopic rates satisfies the following equation: $\frac{w^{+}}{w^{-}}=e^{-\Delta G/RT}$. Therefore the rate $v$ can be rewritten as

|  | $v=Ew^{+}\left( 1-e^{\frac{\Delta G}{RT}} \right)$ | (1) |
| --- | --- | --- |

The terms $w^{+}$ and $w^{-}$ are functions of reactant concentrations and kinetic constants. For instance, for a uni-uni reaction $A\leftrightarrow B$ with mass-action kinetics$v=E\left( k^{+}a-k^{-}b \right)$, we obtain

|  | $v=Ek^{+}a\left( 1-e^{\frac{\Delta G}{RT}} \right)$ | (2) |
| --- | --- | --- |

The reaction rate thus depends on three factors: (i) the enzyme concentration *E*, (ii) the kinetic term $w^{+}=k^{+}a$, and (iii) a function of the thermodynamic force ($\Delta G$) in units of *RT*. The kinetic term in Eq. 3 is only valid for mass-action kinetics. However, other rate laws exist, and the kinetic term can take many different forms (such as in Michaelis-Menten kinetics, allosteric regulation effects, and the “force-dependent modular rate law” [[4](#_ENREF_4)]). Nevertheless, in all of these cases, the thermodynamic term will stay the same. Both terms (kinetic and thermodynamics) depend on the metabolite concentrations, but in different ways.

In order to understand the effect of the reactant concentrations on the reaction rate, we can use the following relationship between the reaction Gibbs energy and the reaction quotient ($Q$) – the concentration ratio between products and substrates, which states that $\Delta G=\Delta G^{\circ}+RT\cdot\ln Q$. Since we also know that $\Delta G^{\circ}=-RT\cdot\ln K_{eq}$ we can rewrite Eq. 2 as: $v=Ew^{+}\left( 1-Q/K_{eq} \right)$. This formulation shows us clearly that while the kinetic term ($w^{+}$) usually increases with the substrate concentrations (in most rate laws), the thermodynamic force term $(1-Q/K_{eq})$ decreases with $Q$.

Thus, Eq. (2) states a general relation between flux and force close to equilibrium [[5](#_ENREF_5)], and assuming a constant kinetic term $w^{+}$, force and flux would be proportional; in reality, the kinetic term may vary, but if it remains in a certain range, the flux will at least tend to increase with the force. We thus use the term $E$in Eq. (2) as the enzyme cost by assuming that it is proportional to the amount of enzyme invested in catalyzing a certain reaction. Explicitly:

|  | $E=\frac{v}{w^{+}\left( 1-e^{\frac{\Delta G}{RT}} \right)}$ | (3) |
| --- | --- | --- |

### Quantitative estimates

To check the prediction from Eq. (4) has the right order of magnitude, let us assume that a flux needs to be realized at a reaction Gibbs energy of $\Delta G=-4RT$ (which is -10 kJ/mol). Taking the flux through a typical enzyme in glycolysis (for *E. coli* growing in mini-chemostats [2]), we set $v= 5 mmol gr\left( cdw \right)^{-1}h^{-1}$. We further assume typical reactant concentrations *c* = 0.1 mM, typical turnover numbers $k_{cat}\approx50 s^{-1}$ (for central metabolic enzymes), typical enzyme weights MW ≈ 30 kDa [[6](#_ENREF_6)] and typical Michaelis constants $K_{M}\approx0.1$ mM [[4](#_ENREF_4)]. For a reversible Michaelis-Menten rate law

|  | $w^{+}=\frac{3600\frac{s}{h}}{MW}\frac{k_{cat}^{+}\frac{a}{K_{M}}}{1+\frac{a}{K_{M}}+\frac{b}{K_{M}}}$ | (4) |
| --- | --- | --- |

we would obtain $w^{+}\approx2000 mmol gr\left( enz. \right)^{-1}h^{-1}$. This yields the estimate

|  | $E=\frac{5\frac{mmol}{gr(cdw)\times h}}{2000 \frac{mmol}{gr(enz.)\times h}\cdot(1-e^{-4})}\approx0.002\frac{gr\left( enz. \right)}{gr(cdw)}$ | (5) |
| --- | --- | --- |

If any of the factors in the calculation changes, for instance, by a factor of 10, $E$ will vary by the same factor. The value 0.2% is reasonable, though, since recent proteomic measurements have shown that the eleven glycolysis enzymes occupy ≈5% of the proteome [[7](#_ENREF_7)], while the proteome comprises ≈55% of the cell dry weight [[8](#_ENREF_8)]. That would mean that, on average, each glycolytic enzyme should occupy about 0.25% of the cell dry weight – which is quite close to the result in Eq. (6). Of course all these estimates are very rough and the value of *w^+^* can vary by several orders of magnitude both between enzymes (e.g., due to the different specific activities) and with the metabolite concentrations.

### Penalty term for small thermodynamic forces

For our concentration predictions, we replace the enzyme cost function shown in Eq.(4) by a simplified penalty function, $I$, in which the factor $\left( 1-e^{\frac{\Delta G}{RT}} \right)^{-1}$ is approximated by a quadratic function. This enables finding an optimal solution using quadratic linear programming. Similarly to $E$, the units of $I$ are $gr\left( enz. \right)\times gr\left( cdw \right)^{-1}$). The formula is thus:

|  | $I\left( v,-\Delta G \right)=\left\{ \begin{matrix} \infty& -\frac{\Delta G}{RT}<\beta\\ v\cdot\left( \gamma_{1}+\gamma_{2}\left( \alpha+\frac{\Delta G}{RT} \right)^{2} \right) & \beta<-\frac{\Delta G}{RT}<\alpha\\ v\cdot\gamma_{1} & \alpha<-\frac{\Delta G}{RT} \end{matrix} \right.$ | (6) |
| --- | --- | --- |

An infinite penalty below a minimal driving force threshold $\beta$ replaces the very large enzyme level at $\Delta G$ values around zero; in practice, these values will be forbidden. As shown in Supp. Figure S2, the ad hoc threshold values and curve parameters: $\beta=0.02$, $\alpha=4$, $\gamma_{1}={10}^{-3} gr\left( enz. \right)\times h\times mmol^{-1}$ and $\gamma_{2}={10}^{-4} gr\left( enz. \right)\times h\times mmol^{-1}$ yield a sensible approximation.

Here, we describe the results obtained with$\beta=0.02$ and $\alpha=4$ while the results are robust for a wide range of parameter choices (see Supp. Figure S4-S5). We note that if specific kinetic constants were available, enzyme-specific values of $\gamma_{1}$ and $\gamma_{2}$ could be incorporated into the penalty function in order to reflect the differences between the enzymes.

It should be noted, that in order to approximate enzyme efficiency based solely on flux rates and metabolite concentrations, we utilize a variant of the enzyme penalty function in Equation (7). Importantly, for the sole purpose of finding the optima for the penalty function $I\left( v,-\Delta G \right)$, we can assume without loss of generality that *ϒ_1_* = 0 and *ϒ_2_* = 1 since their explicit values do not affect the location of the optima for any constant flux *v*. To prove this, it is enough to see that:

$$I\left( v,-\Delta G \right)=v\cdot\gamma_{1}+\gamma_{2}\cdot\tilde{I}(v,-\Delta G)$$

where $\tilde{I}$ is the same as $I$, except that its value of *ϒ_1_* is 0 and *ϒ_2_* is 1, i.e.:

|  | $\tilde{I}=\left\{ \begin{matrix} \infty& -\frac{\Delta G}{RT}<\beta\\ v\cdot\left( \alpha+\frac{\Delta G}{RT} \right)^{2} & \beta<-\frac{\Delta G}{RT}<\alpha\\ 0 & \alpha<-\frac{\Delta G}{RT} \end{matrix} \right.$ | (7) |
| --- | --- | --- |

Since there is an affine transform with positive slope that converts $I$ to $\tilde{I}$, it means that their optima occur at the same values of $\Delta G$.

### Derivation of the formulae

The ratio of the microscopic rates is determined by the reaction Gibbs free energy, $\frac{w^{+}}{w^{-}}=e^{-\Delta G/RT}$ [[5](#_ENREF_5)]. Therefore, the net rate can be written as

|  | $v=E\left( w^{+}-w^{-} \right)=Ew^{+}\left( 1-\frac{w^{-}}{w^{+}} \right)=Ew^{+}\left( 1-e^{\frac{\Delta G}{RT}} \right)$ | (8) |
| --- | --- | --- |

Accordingly, the enzyme level needed to support a given flux $v$ while assuming the reaction is close to equilibrium, i.e. $-1\ll\Delta G<0$, reads

|  | $E=\frac{v}{w^{+}\cdot\left( 1-e^{\frac{\Delta G}{RT}} \right)}\approx\frac{v}{w^{+}\cdot\frac{-\Delta G}{RT}}$ | (9) |
| --- | --- | --- |

Therefore, enzyme level ($E$) and force ($\Delta G$) are inversely proportional (assuming a fixed flux).

## Implementation of mTOW as two sub problems

The formulation of mTOW involves a non-convex mixed-integer optimization problem that is computationally intractable for large-scale networks (methods). Therefore, in order to approximate this formulation using a computationally feasible optimization problem, we utilize the following two-step approach (Supp. Figure S3): (I) identifying a thermodynamically feasible flux distribution under a growth medium at hand. (II) given the flux rates derived from the first step - predicting the optimal metabolite concentrations that satisfy the second law of thermodynamics and that reflect a compromise between the minimization of metabolite and enzyme levels. We show that the prediction performance of mTOW is robust to alternative possible flux distributions predicted in step (I) (Supp. Figure S4).

### mTOW Step I: Predicting a thermodynamically feasible flux distribution

In this step, mTOW aims to identify a thermodynamically feasible flux distribution under a growth medium at hand, utilizing available flux and growth rate measurements. The employed method can be regarded as a variant of Thermodynamic Metabolic Flux Analysis (TMFA) [[9](#_ENREF_9)]. Specifically, we utilize a Mixed-Integer Linear Programming problem to simultaneously search for a flux distribution that satisfies the stoichiometric mass-balance constraint, with a vector of metabolite concentrations that satisfy the second law of thermodynamics (with respect to predicted flux directionality). The predicted set of concentrations is a part of a larger possible solution space possible for the same set of fluxes when constraining thermodynamic feasibility only (as shown in [[10](#_ENREF_10)]). Therefore, the predicted metabolite concentrations arrived at in this step serve only to assure thermodynamic feasibility and are disregarded in subsequent analysis. The formulation of the thermodynamic constraint via a linear equation requires that all reversible reactions in the model are split into two irreversible ones, and additional auxiliary integer variables are used as indicators for which one of the two reactions is active (as descried [[11](#_ENREF_11), [12](#_ENREF_12)]). The explicit formulation for this optimization set is described below.

The measured growth rate was utilized to constrain the biomass production rate (represented as a pseudo-reaction in the model). To identify a genome-scale flux distribution that matches experimental measurements in central metabolism, the objective function is formulated as to minimize the L1 difference between measured and predicted rates for various reactions. To account for potential errors in the standard Gibbs energy data (obtained via CCM), we allowed the ${\Delta G}^{'0}$ values to deviate from the given data, while aiming to minimize the total magnitude of these deviations. The latter step was actually essential for obtaining a thermodynamically feasible reaction flux and metabolite concentration predictions. Accounting for the two optimization criteria, the proximity with measured fluxes and the minimization of deviation from given ${\Delta G}^{'0}$ values, the former objective received the higher priority as it is assumed to rely on a more reliable data source. The overall optimization problem was formulated as following:

$${\mathbf{minimize} \atop\vec{v},\vec{d},ln \left( \vec{c} \right)} \left( \theta\cdot\sum_{i\in known rates} \left| v_{i}-v_{i}^{\mathrm{known}} \right|+\sum_{j\in rxns} \frac{|d_{j}|}{\sigma_{j}} \right)$$

1. $\vec{\Delta G^{'}}=\left( \vec{\Delta{G^{'}}^{\circ}}+\vec{d} \right)+RT\cdot S^{\top}\ln\left( \vec{c} \right)$ Gibbs energy
2. $-\frac{\vec{\Delta G^{'}}}{\mathrm{RT}}\geq\beta$ second law of thermodynamics
3. $S\cdot\vec{v}=0$ Mass balance
4. $d^{L}\leq\vec{d}\leq d^{U}$ Deviation bounds
5. $v_{biomass}=v_{\mathrm{measured}}$ Growth rate
6. $\ln\left( c^{L} \right)\leq\ln\left( \vec{c} \right)\leq\ln(c^{U})$ Concentration bounds

Where Equation #1 calculates the Gibbs energy of reactions. For reactions for which standard Gibbs energy data is available (whose set is denoted by $\vec{{\Delta G}^{'0}}$), the Gibbs energy is computed based on the metabolite concentrations, with *R* and *T* denoting the gas constant ($\frac{kJ}{mol\cdot k}$) and Temperature (K), respectively. Equation #2 enforces our strict requirement that each reaction with a positive flux must have a driving force larger than$\beta$. This equation can be transformed to a linear form via the usage of integer variables (as descried [[11](#_ENREF_11), [12](#_ENREF_12)]). Equation #3 represents stoichiometric mass-balance constraints, with *S* representing a *n* x *m* stoichiometric matrix. Equation #4 sets the bounds on the deviation from the given thermodynamic parameters to account for errors in the standard Gibbs energy values or required violations of the bounds on the concentrations of metabolites. Equation #5 sets the growth rate according to the measurements of [[13](#_ENREF_13), [14](#_ENREF_14)]. Equation #6 restricts metabolite concentrations to a pre-defined range, specifically between 10^-5^ mM and 100 mM. The first term of the objective function represents the optimization criteria of minimal L1 difference between measured and predicted rates for various reactions where $d_{j}$ denote the difference between measured and predicted Gibbs energy of reactions values and $\sigma_{j}$ denote the standard deviation of Gibbs energy of reactions as assessed by CCM. The second term of the objective function represents the minimization of deviations from $\Delta G^{'\circ}$ values. The relative weight of these two objectives is determined by the parameter $\theta$ which is set to 100 to prioritize correct flux prediction. All of the above constants and variables are summarized in Supp. Table S1.

### mTOW Step II: Predicting metabolite concentrations

In this step, mTOW utilizes the identified flux distribution from step I and employs Quadratic programming to predict the metabolite concentrations, by finding the best compromise between minimizing their concentrations and reducing the enzyme cost. Similarly to step I, only thermodynamically feasible solutions that satisfy the second law of thermodynamics are considered.

Minimizing the total sum of metabolite concentrations via a linear or quadratic function is not straightforward in this case, as the optimization problem includes variables that represent the natural log of the concentrations (which are required in order to formulate the second law of thermodynamics as a linear equation). Hence, as an approximation to minimizing the absolute metabolite concentrations, we minimized the squared distance between concentrations and the minimal concentration that is expected for a metabolite on a log scale, such that each metabolite will be penalized according to its concentration (and metabolites with the lowest allowed concentration will not be penalized):

|  | $\tilde{M}\left( c_{i} \right)=\left( \ln\left( \frac{c_{i}}{c^{L}} \right) \right)^{2}$ $\backslash$ | (10) |
| --- | --- | --- |

Where *c_i_* is the predicted concentration for metabolite i, and c^L^ is the minimal concentration value (set to $10 nM$), and *m* is the number of metabolites. Enzyme levels, $\tilde{E\left( c,v \right)}$ was calculated according to eqs. (7):

|  | $\tilde{E}\left( c,v_{j} \right)=\left\{ \begin{matrix} \infty& -\frac{{\Delta G}_{j}}{RT}<\beta\\ v_{j}\cdot\left( \alpha+\frac{{\Delta G}_{j}}{RT} \right)^{2} & \beta<-\frac{{\Delta G}_{j}}{RT}<\alpha\\ 0 & \alpha<-\frac{{\Delta G}_{j}}{RT} \end{matrix} \right.$ | (11) |
| --- | --- | --- |

The complete formulation of the optimization problem is as following:

$${\mathbf{minimize} \atop\log\left( \vec{c} \right)} \sum_{i=1..m} \tilde{M}\left( c_{i} \right)+\delta\cdot\sum_{j\in R_{G}} \tilde{E}\left( c,v_{j} \right))$$

1. $\vec{\Delta G^{'}}=\left( \vec{\Delta{G^{'}}^{\circ}}+\vec{d} \right)+RT\cdot S^{\top}\ln\left( \vec{c} \right)$ Gibbs energy
2. $-\frac{\vec{\Delta G^{'}}}{\mathrm{RT}}\geq\beta$ $v_{j}>0\wedge j\in R_{G}$ second law of thermodynamics
3. $\ln\left( c^{L} \right)\leq\ln\left( \vec{c} \right)\leq ln(c^{U})$ Concentration bounds

Where Equation #1 calculates the Gibbs energy of the reactions, equation #2 enforces our strict requirement that reactions with positive flux will have a driving force larger than some threshold, where $R_{G}$ set contains reactions for which standard Gibbs energy data is available .Equation #3 restricts metabolite concentrations to a pre-defined range as explained above. The first term of the objective function represents the optimization criterion of minimal metabolite load. This is approximated via the minimization of L_2_ distance between the natural log of metabolite concentrations and the log of the minimal allowed concentration. The second term of the objective function represents the minimization of enzyme cost (as explained above), which is quadratic in this step as the flux rates are given as inputs from step I. By changing the value of the weight $\delta$, we utilized the concept of Pareto optimality to explore the tradeoff between the two optimality criteria.

### Robustness analysis of mTOW’s implementation to the specific choice of flux distribution

The first step in mTOW involves the estimation of flux rates under a given growth condition of interest. To assess how the choice of specific flux distributions (from a space of potential solutions) affects the concentration prediction, we performed Flux Variability Analysis (similarly to [[9](#_ENREF_9)]). In this analysis, for each *E. coli* reaction which can carry flux with thermodynamic constraints in glucose media, we predicted two flux rates: one with its maximal flux rate and one with its minimal. We applied the same optimization described in part 4.1, adding the minimization or maximization of reactions as a primary objective (while keeping the other objectives with a smaller weight). For each such flux distribution we first predicted the concentrations and then compared them to measurements by Bennet *et al* [[15](#_ENREF_15)] on aerobic glucose medium. Our results show that in all cases a significant correlation was achieved with an average Pearson correlation of 0.61 (Supp. Figure S4 and Supp. Table S2). In addition, we performed robustness analysis on the values of $\beta$ showing again the the results are robust to a wide range if parameter choosing (Supp. Figure S5).

## Distributed Thermodynamic Bottlenecks forces a concentration gradient

In order for a reaction with a positive standard Gibbs energy to carry flux, a concentration gradient is needed. The same applies to series of consecutive reactions where each has positive standard Gibbs energy. In such cases a decrease in concentration is needed between the initial substrate and final product. The intermediate metabolites typically form a gradient of decreasing concentrations as shown in Figure 1b. This effect becomes more prominent the higher the standard Gibbs energies are.

Here, we calculate an adjusted Gibbs energy ($\Delta G^{'c}$) as the Gibbs energy under physiological cellular concentrations, where all cofactors have certain fixed values (depending on the growth media) and all other reactants are set to 1 mM. Generally, the adjusted Gibbs energy can be written as:

|  | ${\Delta G}^{'c}={\Delta G}^{'0}+RT\left( \sum_{i\in cofactor} n_{i}\ln c_{i}+\sum_{j\in reactant} n_{j}\ln\left( {10}^{-3} \right) \right)$ | (13) |
| --- | --- | --- |

Where ${\Delta G}^{'0}$ is the reaction standard Gibbs energy, $n_{i}$ and $n_{j}$ are the stoichiometric coefficients, and $c_{i}$ are the cofactor concentrations. Given the concentrations of the reactants (and assuming the cofactors have the same fixed concentrations), one can calculate the difference between the adjusted Gibbs energy and the actual Gibbs energy:

|  | ${\Delta G}^{'}-{\Delta G}^{'c}=RT\left( \sum_{j\in reactant} n_{j}\ln\left( c_{j} \right)-\sum_{j\in reactant} n_{j}\ln\left( {10}^{-3} \right) \right)=$ $=RT\sum_{j\in reactant} n_{j}\ln\left( \frac{c_{j}}{{10}^{-3}} \right)$ | (14) |
| --- | --- | --- |

A distributed thermodynamic bottleneck is defined as a series of reactions (a sub-pathway) where the adjusted Gibbs energy is more than 5.7 kJ/mol per reaction (equal to $RT\ln(10))$. We choose this definition since, in such cases, the reactant concentrations will be constrained to have a relatively significant downward gradient. For example, in reactions with only one substrate and one product (besides its cofactors) the ratio between the product and the substrate’s concentrations must be at least 10. To see this, we use the fact that $\Delta G^{'c}>RTln(10)$ and $\Delta G^{'}<0$, so:

|  | $RT\cdot\ln\left( 10 \right)<\Delta G^{'c}-\Delta G^{'}=RT\sum_{j\in reactant} n_{j}\ln\left( \frac{c_{j}}{{10}^{-3}} \right)=$ $=RT\left( \ln\left( \frac{c_{product}}{{10}^{-3}} \right)-\ln\left( \frac{c_{substrate}}{{10}^{-3}} \right) \right)=RT\ln\left( \frac{c_{product}}{c_{substrate}} \right)$ | (14) |
| --- | --- | --- |

therefore, $c_{prodcut}>10\cdot c_{substrate}$. Of course, the larger the adjusted Gibbs energy is, the steeper the gradient needs to be to overcome it.

The distributed thermodynamic bottleneck described on aerobic glucose medium (in the main text) consists of three consecutive reactions from fructose-6P to glycerate-1.3P (the last step is glyceraldehyde-3-phosphate dehydrogenase). Here, the high adjusted Gibbs energy of 21 kJ/mol is describe where: fructose-bisphosphate aldolase (EC 4.1.2.13) has an adjusted Gibbs energy of 8.6 kJ/mol, triose-phosphate isomerase (EC 5.3.1.1) has an adjusted Gibbs energy equals to its ${\Delta G}^{'0}$of 6 kJ/mol (since it has no cofactors and only one substrate and one product), and glyceraldehyde-3-phosphate dehydrogenase (EC 1.2.1.12) has a value of 6.2 kJ/mol.

In the examples of metabolic pathways presented in the main text, we denote as co-factors the following metabolites: ATP, ADP, AMP, Pi, CO2, NAD(H), NADP(H) and PPi.

## The metabolic scope of mTOW predictions

As discussed in the main text, the employed *E. coli* model lacks information on the thermodynamics of Aminoacyl tRNA synthetase that consumes amino-acids. Specifically, the metabolites which are predicted only to participate as substrate of the above reaction (and therefore represented in the biomass reaction) and will be assigned with the minimal allowed concentration are: L-Asparagine, L-Arginine, L-Histidine, L-Valine, L-Tryptophan, L-Proline, L-Phenylalanine, L-Lysine, and L-Isoleucine.

## References

1. Noor, E., et al., *An integrated open framework for thermodynamics of reactions that combines accuracy and coverage.* Bioinformatics, 2012.

2. Feist, A.M., et al., *A genome-scale metabolic reconstruction for Escherichia coli K-12 MG1655 that accounts for 1260 ORFs and thermodynamic information.* Mol Syst Biol, 2007. **3**: p. 121.

3. Liebermeister, W., J. Uhlendorf, and E. Klipp, *Modular rate laws for enzymatic reactions: thermodynamics, elasticities and implementation.* Bioinformatics, 2010. **26**(12): p. 1528-1534.

4. Bar-Even, A., et al., *The moderately efficient enzyme: evolutionary and physico-chemical trends shaping enzyme parameters.* Biochemistry, 2011.

5. Beard, D.A. and H. Qian, *Relationship between Thermodynamic Driving Force and One-Way Fluxes in Reversible Processes.* PLoS ONE, 2007. **2**(1): p. e144.

6. Albe, K.R., M.H. Butler, and B.E. Wright, *Cellular concentrations of enzymes and their substrates.* J. Theor. Biol, 1990. **143**(2): p. 163-195.

7. Lu, P., et al., *Absolute protein expression profiling estimates the relative contributions of transcriptional and translational regulation.* Nature biotechnology, 2006. **25**(1): p. 117-124.

8. Gross, C., et al., *Escherichia coli and Salmonella: cellular and molecular biology.* Escherichia coli and Salmonella: Cellular and Molecular Biology, 1996.

9. Christopher S. Henry, L.J.B., and Vassily Hatzimanikatis, *Thermodynamics-Based Metabolic Flux Analysis.* Biophysical Journal, 2007. **92**: p. 1792-1805.

10. Henry, C.S., L.J. Broadbelt, and V. Hatzimanikatis, *Thermodynamics-based metabolic flux analysis.* Biophys J, 2007. **92**(5): p. 1792-805.

11. Tepper, N. and T. Shlomi, *Predicting Metabolic Engineering Knockout Strategies for Chemical Production: Accounting for Competing Pathways.* Bioinformatics, 2009. **26**(4): p. 536-543.

12. Tepper, N. and T. Shlomi, *Metabolic network-based design of metabolite screening strategies for chemical production. .* submitted, 2010.

13. Bennett, B.D., et al., *Absolute metabolite concentrations and implied enzyme active site occupancy in Escherichia coli.* Nat Chem Biol, 2009. **5**(8): p. 593-9.

14. Amador-Noguez, D., et al., *Metabolome remodeling during the acidogenic-solventogenic transition in Clostridium acetobutylicum.* Applied and Environmental Microbiology, 2011.

15. Bennett BD, *e.a.*, *Absolute metabolite concentrations and implied enzyme active site occupancy in Escherichia coli.* *Nat Chem Biol*, 2009. **5**(8): p. 593-599.

16. Ishii, N., et al., *Multiple High-Throughput Analyses Monitor the Response of E. coli to Perturbations.* Science, 2007. **316**(5824): p. 593-597.

17. Perrenoud, A. and U. Sauer, *Impact of global transcriptional regulation by ArcA, ArcB, Cra, Crp, Cya, Fnr, and Mlc on glucose catabolism in Escherichia coli.* JOURNAL OF BACTERIOLOGY, 2005. **187**(9): p. 3171-3179.

18. Bar-Even, A., et al., *Hydrophobicity and Charge Shape Cellular Metabolite Concentrations.* PLoS Comput Biol, 2011. **7**(10): p. e1002166.

## Supplementary Figures


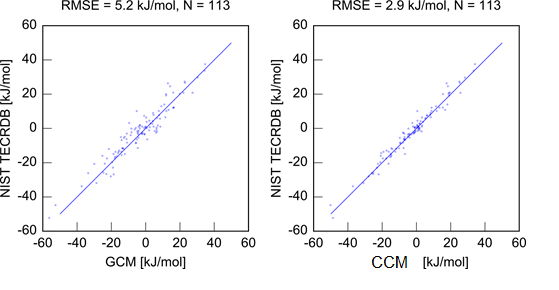


Supp. Figure S1: Evaluation of CCM. CCM results fit the measured data better (RMSE = 2.9 kJ/mol, right panel) than the previous estimations found in iAF1260 (RMSE = 5.2 kJ/mol, left panel). This can be attributed to the fact that the latter are derived from standard GCM which uses the group contributions to estimate the reaction Gibbs energy even when there is explicit data for that reaction in the training set. The reason CCM does not fit the TECRDB exactly, is mainly due to the inconsistencies in the measured reaction database and effects of the uncertainty in pKa values used in the reverse Legendre transform. Since we use the least-squares estimation for CCM, 2.9 kJ/mol is the smallest RMSE that can be achieved if one requires that the reaction energies are consistent with the assumption that each compound has fixed formation energy.


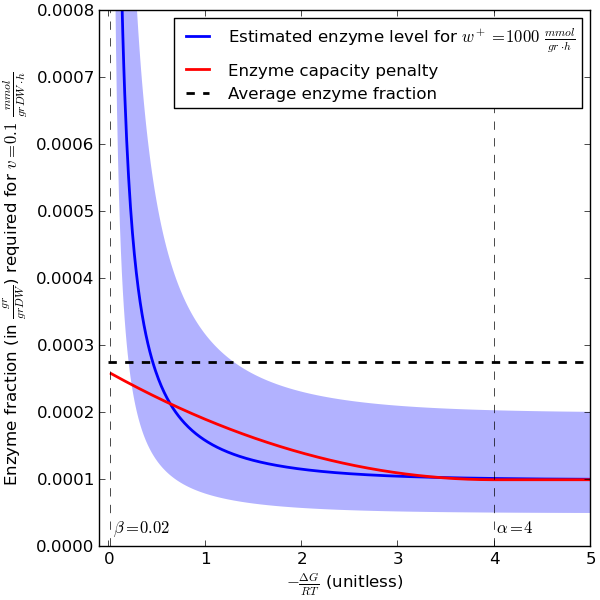


**Supp. Figure S2: Estimated enzyme levels as a function of the thermodynamic driving force −**$\boldsymbol{\Delta G}$**.** The blue line shows necessary enzyme levels (i.e. the required mass fraction out of the total cell dry weight) computed by Eq. (3) with a value w^+^ = 1000 $mmol\times gr\left( enz. \right)^{-1}\times h^{-1}$ and a required flux of 0.1 $mmol\times gr\left( cdw \right)^{-1}\times h^{-1}$, as shown in Eq (6). The band around it shows the range of two-fold changes (assumptions between w^+^ = 500 s^−1^ and w^+^ = 2000 s^−1^). Enzyme levels for a typical-abundance non-ribosomal protein are marked by a dashed line. For flux prediction, we approximate the enzyme level by a penalty term $I$ (red curve, see Eq. 7), which is quadratic between the thresholds $\beta$ and $\alpha$ (vertical dashed lines) and constant above. Parameters are: $\beta=0.02$, $\alpha=4$, $\gamma_{1}={10}^{-3} gr\left( enz. \right)\times h\times mmol^{-1}$ and $\gamma_{2}={10}^{-4} gr\left( enz. \right)\times h\times mmol^{-1}$.


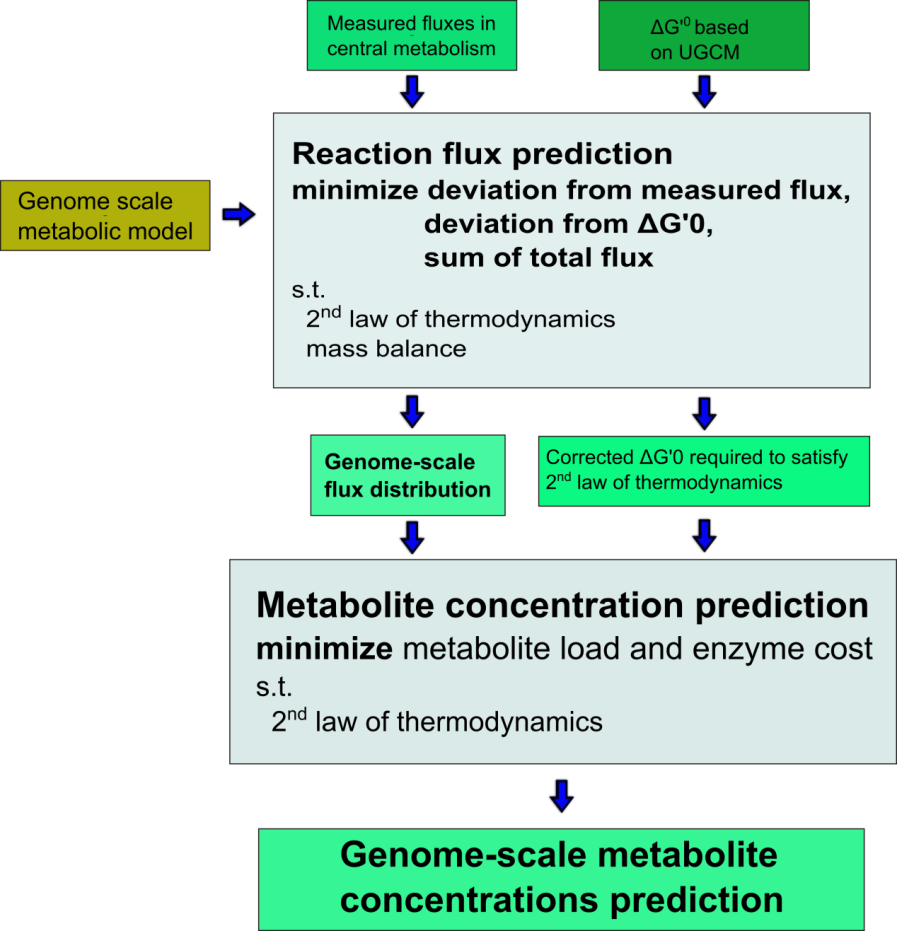


Supp. Figure S3: Workflow for mTOW implementation. We first predict flux rates of reactions flux rates utilizing a genome scale metabolic model and relying on CCM’s $\boldsymbol{\Delta G}^{\boldsymbol{'0}}$ values and measured growth and flux rated. Next, we use the achieved flux distribution and corrected $\boldsymbol{\Delta G}^{\boldsymbol{'0}}$ values to predict metabolite concentrations.


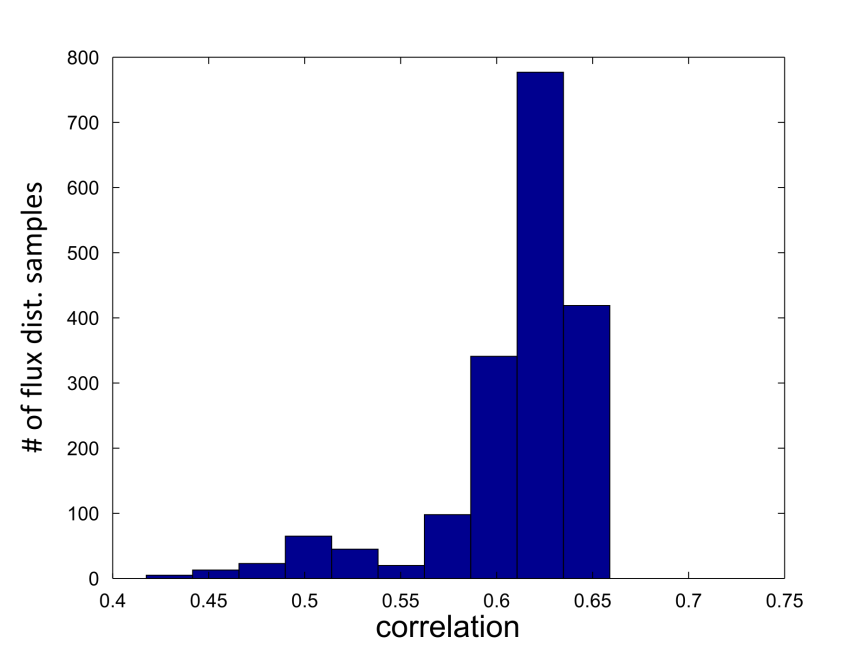


Supp. Figure S4: Distribution of correlation between measured and predicted concentrations according to different flux distributions.


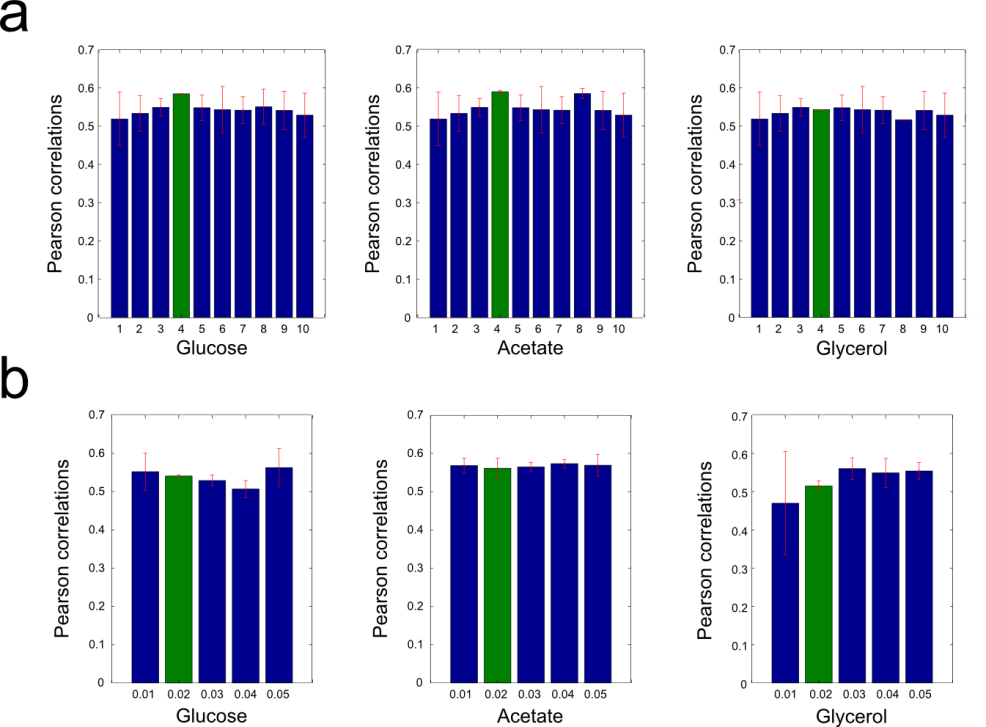


Supp. Figure S5: Distribution of correlation between measured and predicted concentrations according to different values of thermodynamic parameters: (a) alpha; (b)beta. The chosen values for each parameter is marked in green.

Supp. Figure S6: Steady-state labeling patters of ribose-5-phosphate when cells are grown in 1,2-^13^C glucose (100%). Ribose-5-phosphate production via the oxidative pentose phosphate (PP) pathway results in the production of the 1-labeled form of this metabolite, while the 3-labeled form originates from the 1-labeled form due to the reversibility of transketolase and the incorporation of 2-labeled glyceraldehyde-3P. The non-labeled, 2-labeled and 4-labeled forms are produced exclusively from the non-oxidative PP pathway via transketolase and transaldolase. The ratio between the amount of ribose-5P produced via the oxidative vs. the non-oxidative PP pathway is then given by the ratio between the sum of the 1- and 3-labeled forms to the sum of the 0-,2-, and 4-labeled forms. The fraction of ribose-5P produced via the oxidative PP pathway during aerobic and anaerobic conditions is ~85% and 56%, respectively. The data shown represents the average of six replicate experiments in aerobic and anaerobic conditions.

## Supplementary Tables

| Constants | | Variables | |
| --- | --- | --- | --- |
| S | Stoichiometric matrix (n x m) | $v$ | Flux rates (mmol/gr_dw_*h) |
| G_0_ | Gibbs free energy of formation for metabolites (kJ/mol) | $\log\left(c\right)$ | Vector of metabolite (natural) log-concentrations (n x 1) (unitless) |
| R | Gas constant (kJ/mol*K) | $d_{rxn}$ | Vector of deviations in reaction Gibbs energies (m x 1) (kJ/mol) |
| T | Temperature (K) | $\Delta G'$ | Vector of reaction Gibbs energies (m x 1) (kJ/mol) |
| v^known^ | Vector of measured flux rates (m x 1) (mmol/gr_dw_*h)^a^ |  | |
| d^L^, d^U^ | Vector of deviation from Gibbs energy of formation (m x 1) (kJ/mol)^b^ |  |  |
| $\alpha$ | an upper bound on the thermodynamic driving force (RT)^c^ |  |  |
| $\beta$ | Upper bound for Gibbs energy of active reactions (kJ/mol)^d^ |  |  |
| $\mathrm{v}_{\mathrm{measured}}$ | Measured growth rate (mmol/gr_dw_*h)^e^ |  |  |
| c^L^, c^U^ | Upper and lower bounds on metabolites concentrations (M)^f^ |  |  |
| $\theta$ | weight of closeness to flux objective functions^g^ |  |  |
| $\delta$ | Weight of two objective functions (using to compute pareto-optimality front) |  |  |
| $SD_{rxn}$ | Standard deviation of Gibbs energies (kJ/mol) |  |  |

Supp. Table S1: Constants and variables in mTOW’s implementation

^a^Flux rates were taken from the measurements of [[14](#_ENREF_14), [16](#_ENREF_16)] on aerobic glucose-fed *E. coli*, [[17](#_ENREF_17)] on anaerobic glucose-fed *E. coli*, and [[14](#_ENREF_14)] for *C. acetobutylicum*.
 ^b^The Gibbs energies were bounded to deviate by no more than twice the standard deviation.
^c^$\alpha=4$ (corresponding to a $\Delta G$ of -10 kJ/mol
^d^The upper bound for Gibbs energy of active reactions was set to -0.05 kJ/mol (=-0.02RT)
^e^Growth rates were set according to Bennett et al [[15](#_ENREF_15)] on aerobic *e. coli* media and by [[14](#_ENREF_14)] on c. *acetobutylicum* (mmol/gr(cdw)*h)
^f^The upper concentration bound value was set to 100 mM and lower concentration bound to 10^-5^ mM. In addition, concentrations of metabolites in the medium and currency metabolites were set: (i) Nutrients in growth medium (as set by Henry *et al* [[9](#_ENREF_9)]): P_i_ was set to 56 mM, sulfate to 0.3 mM, ammonium to 1.9 M, sodium to 0.16 M, potassium to 2.2 mM, iron to 6.2 mM, and CO_2_ to 0.01 mM. (ii) Oxygen was set as following: extracellular to $8.2\cdot{10}^{-6}$ M, periplasm concentration was set between 10^-7^ M and $8.2\cdot{10}^{-6}$ M, and cytoplasm concentration between 10^-7^ M and $8.2\cdot{10}^{-6}$ M (iii) pH was set to 7 (iv) The concentrations of ATP, ADP, AMP, NAD^+^, NADH, and P_i_ currency metabolites were set according to the different measured datasets ( [[15](#_ENREF_15)] for *E. coli* in glucose, acetate and glycerol media and; the measurements on anaerobic conditions for *E. coli*; [[14](#_ENREF_14)] for *C. acetobutylicum*)_i_.
^g^The weight of the objective function were set to $\theta$ = 100

| Spearman correlations for log [predicted concentration] vs. log [measured concentration] | *E. coli* | | | | *C. acetobutylicum* | |
| --- | --- | --- | --- | --- | --- | --- |
|  | Aerobic Glucose | Aerobic Acetate | Aerobic Glycerol | Anaerobic glucose | Acido- genesis | Solveno-genesis |
| Chemical properties [[18](#_ENREF_18)] | 0.54  $(p<{10}^{-7})$ | 0.42  $(p<{10}^{-3})$ | 0.46  $(p<{10}^{-3})$ | Not significant | 0.3  $(p<{10}^{-2})$ | Not significant |
| **mTOW** | **0.58**  $(p<{10}^{-5})$ | **0.66**  $(p<{10}^{-4})$ | **0.67**  $(p<{10}^{-4})$ | **0.71**  $(p<{10}^{-4})$ | **0.46**  $(p<{10}^{-4})$ | **0.45** $(p<{10}^{-3})$ |

Supp. Table S2: Spearman correlation between predicted and measured metabolites concentrations

|  |  | *E. coli* Aerobic glucose | *E. coli* Aerobic Acetate | *E. coli* Aerobic Glycerol |
| --- | --- | --- | --- | --- |
|  |  |  |  |  |
| Correlation in chosen Pareto solution | | **0.59**  $(p<{10}^{-5})$ | **0.61**  $(p<{10}^{-4})$ | **0.55**  $(p<{10}^{-4})$ |
| Maximal decrease in correlation | | 0.5  (15% decrease) | 0.58  (5% decrease) | 0.52  (5.5% decrease) |
| Average decrease in correlation | | 0.55  (7% decrease) | 0.58  (5% decrease) | 0.53  (5% decrease) |

**Supp. Table S3:** Examining the set of Pareto optimal solutions (per media) in which each of the objectives deviates in not more than 20% from the chosen solution)

|  |  | *E. coli* Aerobic glucose | *E. coli* Aerobic Acetate | *E. coli* Aerobic Glycerol |
| --- | --- | --- | --- | --- |
|  |  |  |  |  |
| Integrating mTOW and chemical properties prediction vs. measured concentrations [[13](#_ENREF_13)] | | 0.91 | 0.8 | 0.78 |
| Comparing top of the 95% confidence intervals range of measured concentrations to the reported values [[13](#_ENREF_13)] | | 0.38 | 0. 68 | 0.46 |

**Supp. Table S4:** Evaluation of mTOW’s prediction errors in comparison to experimental measurement error (in terms of RMSE scores)
